# TGFβ pathway is required for viable gestation of Fanconi anemia embryos

**DOI:** 10.1371/journal.pgen.1010459

**Published:** 2022-11-28

**Authors:** Alfredo Rodríguez, Michael Epperly, Jessica Filiatrault, Martha Velázquez, Chunyu Yang, Kelsey McQueen, Larissa A. Sambel, Huy Nguyen, Divya Ramalingam Iyer, Ulises Juárez, Cecilia Ayala-Zambrano, David B. Martignetti, Sara Frías, Renee Fisher, Kalindi Parmar, Joel S. Greenberger, Alan D. D’Andrea

**Affiliations:** 1 Department of Radiation Oncology, Dana Farber Cancer Institute, Boston, Massachusetts, United States of America; 2 Departamento de Medicina Genómica y Toxicología Ambiental, Instituto de Investigaciones Biomédicas, Universidad Nacional Autónoma de México, Ciudad Universitaria, México, México; 3 Instituto Nacional de Pediatría, Mexico City, Mexico; 4 University of Pittsburgh Medical Center, Pittsburgh, Pennsylvania, United States of America; 5 Center for DNA Damage and DNA Repair, Dana Farber Cancer Institute, Boston, Massachusetts, United States of America; 6 Posgrado en Ciencias Biológicas, UNAM, Ciudad Universitaria, México, México; Cornell University, UNITED STATES

## Abstract

Overexpression of the TGFβ pathway impairs the proliferation of the hematopoietic stem and progenitor cells (HSPCs) pool in Fanconi anemia (FA). TGFβ promotes the expression of NHEJ genes, known to function in a low-fidelity DNA repair pathway, and pharmacological inhibition of TGFβ signaling rescues FA HSPCs. Here, we demonstrate that genetic disruption of *Smad3*, a transducer of the canonical TGFβ pathway, modifies the phenotype of FA mouse models deficient for *Fancd2*. We observed that the TGFβ and NHEJ pathway genes are overexpressed during the embryogenesis of *Fancd2*^*-/-*^ mice and that the *Fancd2*^-/-^*Smad3*^*-/-*^ double knockout (DKO) mice undergo high levels of embryonic lethality due to loss of the TGFβ-NHEJ axis. Fancd2-deficient embryos acquire extensive genomic instability during gestation which is not reversed by Smad3 inactivation. Strikingly, the few DKO survivors have activated the non-canonical TGFβ-ERK pathway, ensuring expression of NHEJ genes during embryogenesis and improved survival. Activation of the TGFβ-NHEJ axis was critical for the survival of the few *Fancd2*^-/-^*Smad3*^*-/-*^ DKO newborn mice but had detrimental consequences for these surviving mice, such as enhanced genomic instability and ineffective hematopoiesis.

## Author summary

The TGFβ pathway inhibits the growth and proliferation of the hematopoietic stem and progenitor cells in Fanconi anemia (FA). We have previously shown that inhibition of the TGFβ pathway rescues the growth defects of FA hematopoietic stem and progenitor cells. In this work we determined whether a cross between *Fancd2* (an FA gene) and *Smad3* (the canonical TGFβ pathway transducer) heterozygous mice would generate double knockout (*Fancd2*^*-/-*^*Smad3*^*-/-*^*)* progeny without the hematopoietic defect characteristic of FA. Contrary to expected, we observed increased embryonic lethality for the double knockout (DKO) mice and persistence of the FA phenotype in the surviving DKO mice. We discovered that *Fancd2*^*-/-*^ embryos acquire extensive genomic instability during gestation which is not reversed by *Smad3* inactivation. In addition, the *Fancd2*^*-/-*^ mouse embryos exhibited high NHEJ activity which allowed them to survive during gestation. The few DKO survivors retain the FA phenotype due to activation of the non-canonical TGFβ -ERK pathway.

## Introduction

Fanconi anemia (FA) is a chromosome instability syndrome with childhood onset bone marrow failure (BMF), congenital malformations, and cancer predisposition [1]. A defect in the maintenance of the hematopoietic stem and progenitor cell (HSPC) pool in FA patients leads to BMF and aplastic anemia (AA). FA is a recessive disease caused by pathogenic variants inherited in any one of 22 *FANC* genes [2]. The protein products of the *FANC* genes participate in a biochemical pathway involved in the repair of DNA interstrand crosslinks (ICLs) [3]. A defect in the FA pathway results in hypersensitivity to ICL-inducing agents, such as acetaldehyde, formaldehyde, mitomycin C (MMC), or diepoxybutane (DEB). Acetaldehyde and formaldehyde are currently considered to be the source of endogenous DNA damage, causing HSPC attrition in the BM of FA patients [4].

Accumulation of DNA damage in FA HSPCs leads to several cellular defects. On the one hand, hyperactivation of p53 in FA cells blocks progression into the cell cycle and promotes apoptosis [5]. On the other hand, overexpression of the oncogene *MYC* in FA HSPCs provides a growth-promoting compensatory mechanism, counteracting p53 and supporting the survival of the limited HSPC pool in the FA bone marrow [6].

Proinflammatory cytokines are upregulated in the FA bone marrow microenvironment of FA patients [7–10]. Increased TNFα expression activates MYC upregulation [6], and increased TGFβ1 and TGFβ3 has growth suppressive activity [7]. The TGFβ proinflammatory cytokines belong to a superfamiliy of multifunctional ligands with the capacity to signal into several receptors [11]. The outcomes of TGFβ signaling have been shown to be context dependent. TGFβ1 and TGFβ3 suppress the growth of HSPCs, whereas TGFβ2 has an opposite role and promotes hematopoiesis [12]. In HSPCs, the TGFβ family ligands signal through the TGFβ receptor 1 (TGFβ RI), which activates the canonical TGFβ signal transduction pathway, mediated by SMAD3. SMAD3 in turn heterodimerizes with SMAD4 and functions as a transcription factor with the capacity to activate multiple genes [13]. The TGFβ family ligands can also exert their activity through multiple non-canonical pathways that transduce the extracellular signaling into the intracellular milieu, which includes the ERK pathway [14].

The TGFβ pathway has a role in controlling the expression of DNA repair genes [15–18]. Specifically in FA, the increased levels of TGFβ signaling through the canonical SMAD3 pathway promotes the transcriptional upregulation of NHEJ genes and downregulation of HR genes. [10]. The NHEJ pathway is a high capacity but compromised fidelity DNA repair pathway [19] which, when overactivated, can lead to gross chromosomal abnormalities [20]. Importantly, pharmacological inhibition of the TGFβ pathway improves the DNA repair capacities of FA cells by inhibiting the expression of NHEJ genes and increasing the expression of homologous recombination (HR) genes [10]. TGFβ pathway inhibitors thereby promote the survival and growth of FA HSPCs from FA patients *in vitro* and in FA mouse models with physiologically-induced DNA damage [7, 10]. Although pharmacological inhibition of the TGFβ pathway is beneficial for FA HPSCs, rescuing several of the FA HSPCs phenotypes, this may not be the case when the TGFβ pathway is genetically abrogated at the organismal level [13].

Here, we generated a double knockout (DKO) murine model for *Fancd2* and the canonical TGFβ pathway gene *Smad3 (Fancd2*^*-/-*^*Smad3*^*-/-*^*)*, and thereby explored the role of the TGFβ pathway during FA embryogenesis. We observed that, *Fancd2*^*-/-*^ embryos overexpress many components of the TGFβ and NHEJ pathways and complete pregnancy; however, *Fancd2*^*-/-*^*Smad3*^*-/-*^ DKO presented high levels of embryonic lethality. Strikingly, the small fraction of surviving DKO pups retain the characteristic FA phenotypes, including ICL sensitivity and bone marrow failure. Surviving DKO pups exhibited activation of the non-canonical TGFβ-ERK pathway and expression of NHEJ genes, such as DNA-PKcs, demonstrating that the TGFβ-NHEJ axis is required for viable gestation of FA embryos. Strikingly two biochemically distinct types of DKO embryos were identified during early gestation (day E12.5). The first type loses activation of the TGFβ-SMAD3-DNA-PKcs axis, as originally predicted, and the second type exhibits activation of the TGFβ-ERK-DNA-PKcs axis, as observed in the surviving DKO pups.

In summary, we build on the fact that the TGFβ network is very robust and highly connected. We first show a synthetic lethal interaction between the FA pathway and the TGFβ-NHEJ axis during embryogenesis, i.e. *Fancd2*^*-/-*^ embryos need the TGFβ-SMAD3-NHEJ axis for completing embryogenesis. Interestingly, and shown in this manuscript, activation of the ERK pathway, can rescue *Fancd2*^*-/-*^ embryos from the synthetic lethality by activating the non-canonical TGFβ-ERK-NHEJ axis. Our findings demonstrate that the TGFβ pathway is essential for expression of NHEJ genes during FA embryogenesis. Although NHEJ genes are required for viable gestation of FA embryos, NHEJ upregulation is detrimental for hematopoiesis of the embryos and the newborn mice.

## Results

### TGFβ pathway inhibitors rescue hematopoiesis in FA models

**Fig 1:**
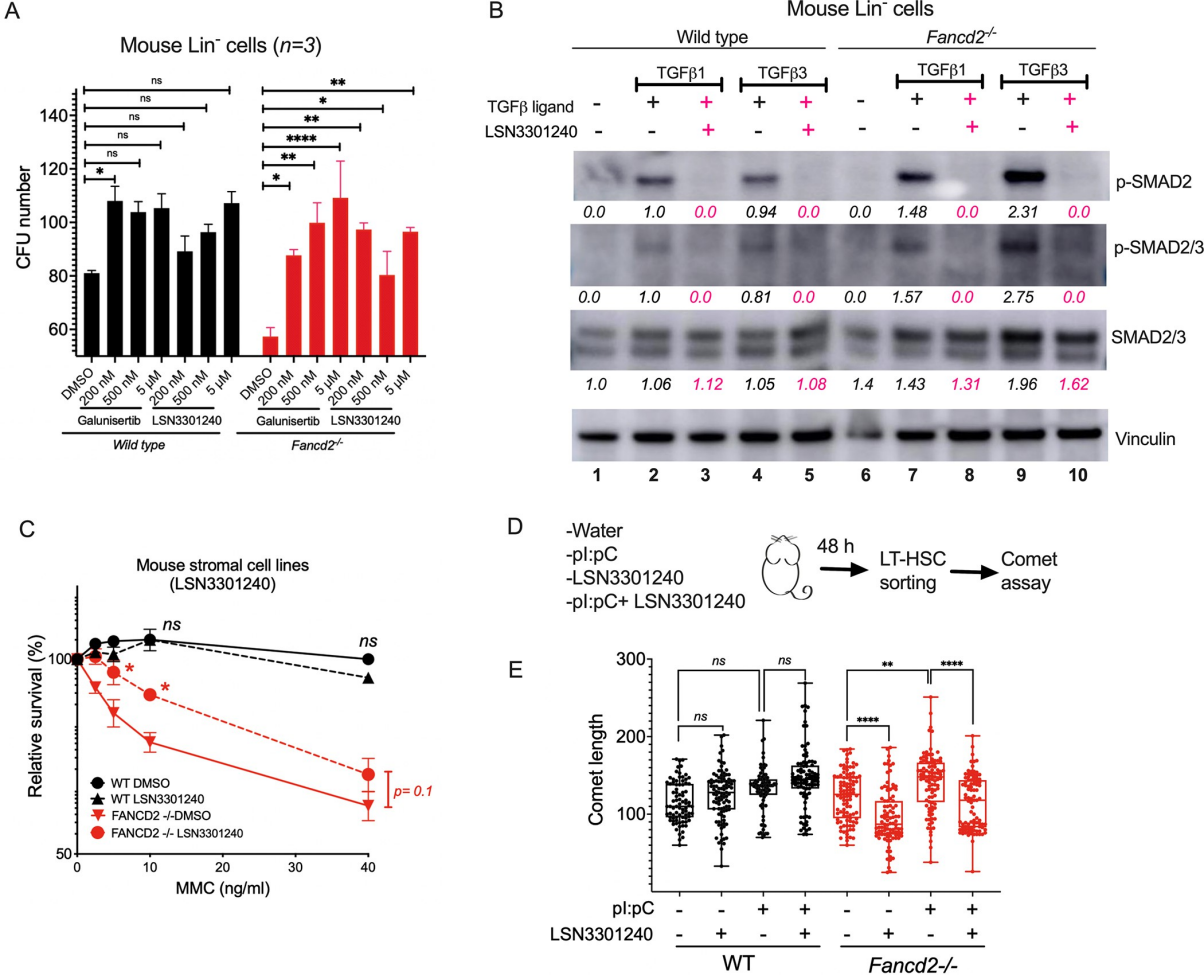
TGFβ pathway inhibitors rescue hematopoiesis in FA models. **(A)** TGFβ pathway inhibitors Galunisertib and LSN3301240 increase the clonogenic capacity of Lin^-^ cells from bone marrow of *Fancd2*^*-/-*^ mice measured in a CFU assay. Lineage negative primary bone marrow cells from wild-type (WT) or *Fancd2*^*-/-*^ mice were cultured in methylcellulose medium containing increasing doses of Galunisertib or LSN3301240 for 7 days and hematopoietic colonies (CFUs) were counted for evaluating clonogenic growth of progenitors. **(B)** Western blots of the lysates from Lin^−^ cells from bone marrow of wild-type and *Fancd2*^*-/-*^ mice cultured for 2 hours in the presence of TGFβ1 (5 ng/mL) or TGFβ3 (5 ng/mL) with or without LSN3301240. Levels of phospho-Smad2 (measured by two different antibodies, one against phospho-Smad2 and the other against both phospho-Smad2 and Smad3) are shown. Quantification relative to basal conditions and the loading control is shown below every lane. Pink indicates samples treated with LSN3301240. **(C)** Stromal cell lines generated from WT and *Fancd2*^*-/-*^ mice were cultured in the presence of LSN3301240 and Mitomycin C (MMC) and survival was determined. LSN3301240 partially rescued the MMC sensitivity characteristic of FA cell lines. **(D)** Schematics showing treatment of WT and *Fancd2*^*-/-*^ mice *in vivo* with pI:pC + LSN3301240. Wild-type (WT) or *Fancd2*^*-/-*^ mice (KO) were injected with pI:pC along with LSN3301240 and 48 hrs after the exposure, DNA damage was analyzed in bone marrow LT-HSCs using a comet assay. **(E)** Comet assay on sorted LT-HSCs showing that pI:pC increases the DNA damage, measured by tail length, of LT-HSCs, whereas co-treatment with LSN3301240 reduces DNA damage in LT-HSCs from *Fancd2*^*-/-*^ mice. Data in (A) and (C) are represented as mean ± SEM. Data in (E) are represented as boxplots. p values of 0.01 to 0.05 were considered significant (*), p values of 0.001 to 0.01 were considered very significant (**) and p values of < 0.001 were considered extremely significant (***, ****). See also S1 Fig.

The canonical TGFβ pathway is upregulated in the bone marrow of patients with FA [7, 10]. Accordingly, TGFβ pathway inhibitors, targeting the TGFβ Receptor I (TGFβ RI), such as Galunisertib and LSN3301240, were initially tested for their ability to rescue hematopoiesis in FA models. As predicted, these inhibitors increased the *in vitro* colony forming unit (CFU) clonogenic capacity of Lin^-^ cells from *Fancd2*^*-/-*^ mice (**Fig 1A**), correlating with a decrease in the phosphorylation of SMAD2/3 in the presence of the TGFβ1 and TGFβ3 in WT and *Fancd2*^*-/-*^ cells (**Fig 1B**). The inhibitors also increased the CFU numbers in the cells from WT mice, however, the increase in CFU was more significant in cells from *Fancd2*^*-/-*^ mice. Consistent with previous studies with other TGFβ pathway inhibitors [7, 10], Galunisertib and LSN3301240 also improved the clonogenic capacity of FA-like HSPCs. These FA-like cells were generated by infection of CD34^+^ cord blood cells with a lentivirus expressing a shRNA against *FANCD2* (**S1A Fig**). LSN3301240 also partially rescued *Fancd2*^*-/-*^ stromal cell lines from MMC-induced genotoxicity (**Fig 1C**), although this effect was not observed with Galunisertib (**S1B Fig**). We next tested LSN3301240 for its ability to protect LT-HSC from DNA damage caused by pI:pC, an agent known to induce physiologic stress [21]. We co-injected WT or *Fancd2*^*-/-*^ mice with pI:pC and LSN3301240, and the amount of DNA damage in sorted LT-HSC (identified as Lin^-^LSK CD150^+^CD48^-^ cells) was quantified using the comet assay (**Fig 1D**). Treatment of *Fancd2*^*-/-*^ mice with LSN3301240 significantly reduced the amount of DNA damage induced by pI:pC in LT-HSCs (**Fig 1E**). Taken together, inhibition of the TGFβ pathway rescued several of the hematopoietic features of FA bone marrow cells.

### Double knockout of the canonical TGFβ pathway and *Fancd2* results in embryonic lethality

**Fig 2:**
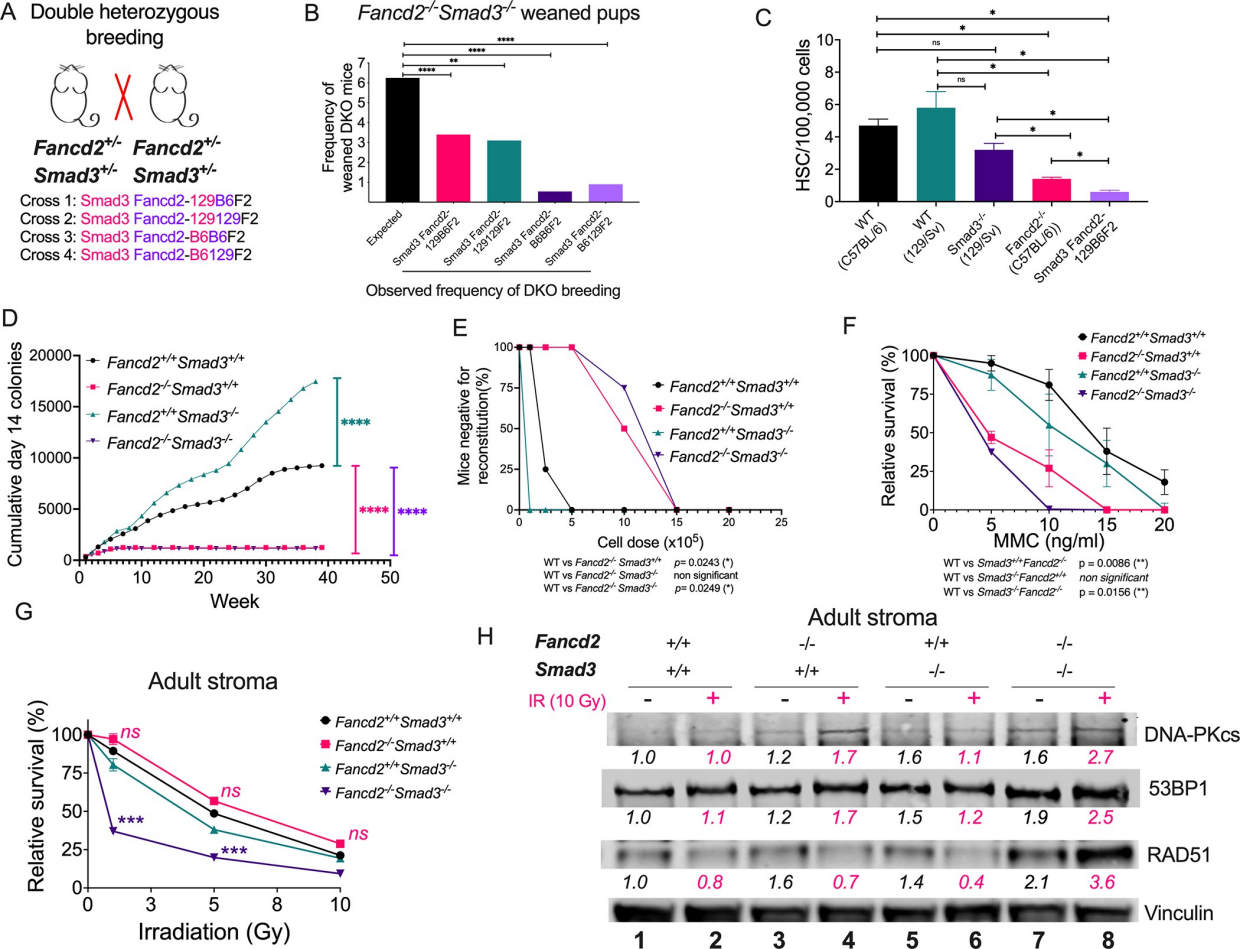
*Smad3* loss is synthetic lethal with *Fancd2* loss and surviving DKO mice do not have improved adult hematopoiesis. **(A)** Two different *Smad3*^*+/-*^ and *Fancd2*^*+/-*^ mouse strains on two different genetic backgrounds (129/Sv and C57BL/6) were bred to obtain 4 strains of *Smad3*^*-/-*^ *Fancd2*^*-/-*^ double Knockout (DKO) mice: Smad3 Fancd2-129B6F2; Smad3 Fancd2-129129F2; Smad3 Fancd2-B6B6F2 and Smad3 Fancd2-B6129F2 (See also S2A Fig). **(B)** Breeding frequency of DKO mice was significantly reduced in all 4 background combinations compared to the expected 6.5% frequency of DKO births from breeding double heterozygotes, suggesting embryonic lethality. For Smad3 Fancd2-129B6F2; Smad3 Fancd2-129129F2; Smad3 Fancd2-B6B6F2 and Smad3 Fancd2-B6129F2, we tested whether the frequency of births is significantly different from an expected frequency of 1 in 16, using the two-sided proportional test. The p-values are adjusted for multiple tests with the Bonferroni method. The adjusted p-values for the four breeds are <0.0001, 0.0028, <0.0001 and 0.0044 respectively, so the birth frequencies are all significantly smaller than 1/16 (i.e. 0.0625). The exact 95% confidence interval for the four breeds are respectively (0.0251, 0.0462), (0.0187, 0.0468), (0.0006, 0.0192) and (0.0011, 0.0325). **(C)** Reduced frequency of bone marrow hematopoietic stem cell (HSC) numbers in Smad3 Fancd2-129B6F2 mice compared to parental strains. **(D)** *In vitro* LTBMC assay showing that bone marrow from adult Smad3 Fancd2-129B6F2 (*Fancd2*^*-/-*^*Smad3*^*-/-*^) mice have a reduced production of hematopoietic progenitors, similar to a *Fancd2*^*-/-*^ (*Fancd2*^*-/-*^ *Smad3*^*+/+*^) mouse bone marrow genotype. Results are presented as cumulative day 14 CFU-GEMM forming cells. **(E)** Competitive repopulation capacity of the bone marrow in transplant assays showing that hematopoietic cells derived from adult Smad3 Fancd2-129B6F2 (*Fancd2*^*-/-*^*Smad3*^*-/-*^) mice have a defective hematopoiesis, similar to *Fancd2*^*-/-*^ (*Fancd2*^*-/-*^ *Smad3*^*+/+*^) mice. **(F)** Survival of stromal cell lines derived from the bone marrow of adult mice showing that cells from Smad3 Fancd2-129B6F2 (*Fancd2*^*-/-*^*Smad3*^*-/-*^) mice are hypersensitive to MMC. **(G)** Survival of stromal cell lines derived from bone marrow of adult mice showing that cells from Smad3 Fancd2-129B6F2 (*Fancd2*^*-/-*^*Smad3*^*-/-*^) mice are hypersensitive to irradiation. **(H)** Western blots of the lysates from stromal cell lines derived from bone marrow of adult mice showing that cells from Smad3 Fancd2-129B6F2 (*Fancd2*^*-/-*^*Smad3*^*-/-*^) mice overexpress the NHEJ proteins DNA-PKcs and 53BP1, and RAD51 a mediator for homologous recombination. A representative blot from three independent experiments is shown. Quantification relative to wild type in basal conditions and the loading control is shown below every lane. Pink indicates irradiated samples (10 Gy). Cell lysis for western blot was performed 4 h after IR. Data in (B) and (C) are represented as bar plots. Data in (D), (E), (F) and (G) are represented as mean ± SEM. p values of 0.01 to 0.05 were considered significant (*), p values of 0.001 to 0.01 were considered very significant (**) and p values of < 0.001 were considered extremely significant (***, ****). See also S2 Fig.

Hyperactive TGFβ pathway signaling causes overexpression of genes in the low-fidelity NHEJ pathway of DNA repair, thereby contributing to the bone marrow dysfunction observed in FA mice and FA patients. Since pharmacological inhibition of the TGFβ pathway improves bone marrow dysfunction, we hypothesized that a mouse model with disruption of *Fancd2* and *Smad3*, the canonical mediator of the TGFβ pathway, would overcome this FA hematopoietic defect and perhaps *increase* the survival of DKO newborn mice. To test this hypothesis, we generated four different double knockout (DKO) crosses of *Smad3*^*+/-*^ and *Fancd2*^*+/-*^ mouse strains. Two different *Smad3*^*+/-*^ and *Fancd2*^*+/-*^ mice strains on two different genetic backgrounds (129/Sv and C57BL/6) were bred to obtain 4 strains of *Smad3*^*-/-*^ *Fancd2*^*-/-*^ DKO mice, named Smad3 Fancd2-129B6F2;

Smad3 Fancd2-129129F2; Smad3 Fancd2-B6B6F2 and Smad3 Fancd2-B6129F2 (**Figs 2A and S2A**). Phenotype for these crosses can be seen in **S1 Table.**

Surprisingly, for every tested DKO breed we observed a *decrease* in the number of weaned DKO pups (**Fig 2B**), demonstrating that embryonic lethality was characteristic of DKO embryos, regardless of the mouse strain. These findings suggested that overexpression of the TGFβ pathway is paradoxically *required* for the survival of embryos with a FA pathway deficiency. Since the Smad3 Fancd2-129B6F2 cross yielded the highest number of surviving DKO mice we analyzed the hematopoietic function of Smad3 Fancd2-129B6F2 adult mice.

### Surviving DKO mice exhibit dysfunctional adult hematopoiesis

Smad3 Fancd2-129B6F2 adult mice were next evaluated for their hematopoietic function, compared to WT, *Fancd2*^*-/-*^, and *Smad3*^*-/-*^ adult mice. The Smad3 Fancd2-129B6F2 adult mice and the *Fancd2*^*-/-*^ mice exhibited a reduced number of hematopoietic stem cells, compared to the WT and Smad*3*^*-/-*^ mice (**Fig 2C).** Similarly, hematopoietic stem cells from adult Smad3 Fancd2-129B6F2 mice or *Fancd2*^*-/-*^ mice were impaired in their production of hematopoietic progenitors, based on LTBMC (Stroma-dependent long-term bone marrow culture) assays (**Fig 2D**), compared with the WT and *Smad3*^*-/-*^ mice. Similar results were observed in LTBMC assays for the Smad3 Fancd2-129129F2; Smad3 Fancd2-B6B6F2 and Smad3 Fancd2-B6129F2 crosses (**S2B Fig**). *In vivo* repopulation assays also revealed a reduced repopulation capacity of Smad3 Fancd2-129B6F2 and *Fancd2*^*-/-*^ hematopoietic stem cells (**Fig 2E**). A similar reduction in repopulation capacity was observed for hematopoietic cells derived from Smad3 Fancd2-129129F2 mice (**S2C Fig**).

*In vitro* assessment of DNA repair capacity in stromal fibroblast lines demonstrated that Smad3 Fancd2-129B6F2 cells are hypersensitive to MMC, similar to the phenotype of *Fancd2* deficient cells (**Fig 2F**), with modest differences likely due to their genetic backgrounds, Smad3 Fancd2-129129F2; Smad3 Fancd2-B6B6F2 and Smad3 Fancd2-B6129F2 derived stroma were also sensitive to MMC (**S2D Fig**). These results were also surprising since *Fancd2*-deficient cells exposed to the TGFβ pathway inhibitors (Galunisertib or LSN3301240), had relative MMC resistance (**Fig 1C**). Smad3 Fancd2-129B6F2 cells were also hypersensitive to irradiation (**Fig 2G),** implying that Smad3 Fancd2-129B6F2 cells have multiple DNA repair defects.

We hypothesized that the sensitivity to irradiation in the Smad3 Fancd2-129B6F2 cells is due to deficiency in the expression of NHEJ proteins; however, after irradiating the cells we observed that Smad3 Fancd2-129B6F2 cells express DNA-PKcs and 53BP1, the prototypical proteins mediating NHEJ (**Fig 2H**). Smad3 Fancd2-129B6F2 cells not only had upregulation of NHEJ proteins but also expressed high levels of RAD51, a protein known to mediate HR (**Fig 2H**). Nonetheless, the DKO cells were unable to tolerate IR.

### Upregulation of TGFβ pathway genes and error-prone DNA repair pathway genes in FA mouse embryos

**Fig 3:**
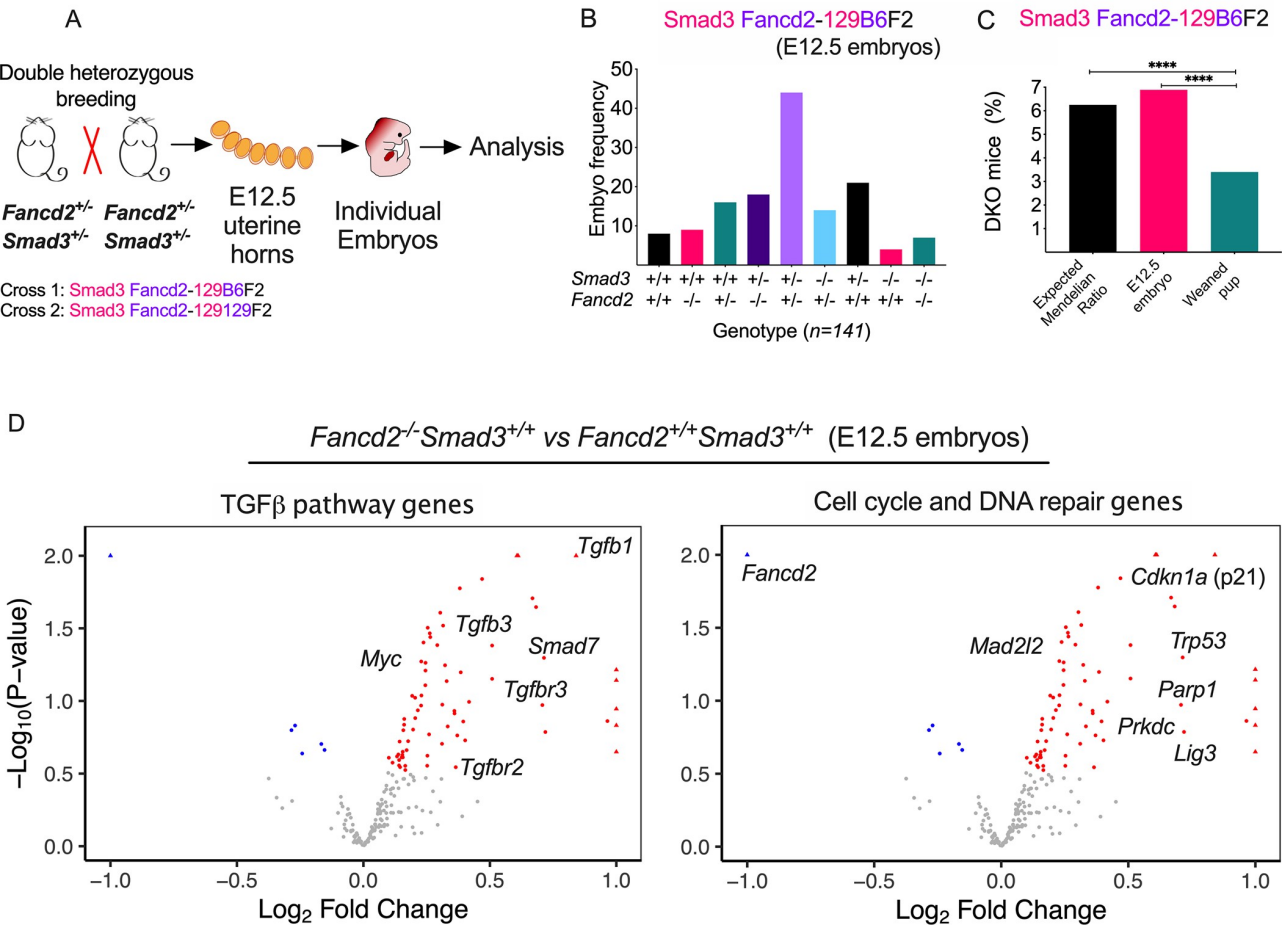
*Fancd2*^*-/-*^ embryos overexpress the TGFβ pathway and error-prone DNA repair genes. **(A)** *Fancd2/Smad3* heterozygous littermates were crossed to breed. Pregnant females were sacrificed at day E12.5 of gestation, uterine horns were dissected, and embryos genotyped. RNA from embryos was analyzed for expression of genes in the TGFβ pathway and DNA repair pathways. **(B)** Smad3 Fancd2-129B6F2 breeding has a Mendelian distribution at day E12.5, showing that Smad3 Fancd2-129B6F2 embryos can survive until day E12.5 of embryonic development (*n = 141 embryos*). **(C)** The frequency of Smad3 Fancd2-129B6F2 embryos at day E12.5 yields the expected Mendelian ratio, however the frequency of weaned Smad3 Fancd2-129B6F2 pups is halved, suggesting later lethality during embryonic development. **(D)** Gene expression analysis of E12.5 embryos. **Left**. Compared to WT embryos, *Fancd2*^*-/-*^ embryos overexpress TGFβ pathway-related genes and overexpress the ligands for activation of the TGFβ pathway, *Tgfb1* and *Tgfb3*, as well as their receptors *Tgfbr2* and *Tgfbr3*. The oncogene *Myc* was also found to be overexpressed in *Fancd2*^*-/-*^ embryos. **Right**. Compared to WT embryos, *Fancd2*^*-/-*^ embryos overexpress genes related to alternative DNA repair pathways, including *Prkdc*, *Mad2l2* and *Parp1*, as well as negative regulators of cell cycle progression, including *Trp53* (p53) and *Cdkn1a* (p21). Red dots indicate differentially upregulated genes with respect to wild type embryos. Blue dots indicate differentially downregulated genes with respect to wild type embryos. Data in (B) and (C) are represented as bar plots. Data in (D) are represented as volcano plots. p values of 0.01 to 0.05 were considered significant (*), p values of 0.001 to 0.01 were considered very significant (**) and p values of < 0.001 were considered extremely significant (***, ****). See also S3 Fig.

We hypothesized that the few surviving DKO pups were the exception to the generalized lethality observed for the DKO crosses. We reasoned that the canonical TGFβ pathway might have a critical role in allowing *Fancd2*^*-/-*^ embryos to survive during mid-gestation. To test this hypothesis, pregnant females were sacrificed at day E12.5, uterine horns were dissected, and the recovered embryos were genotyped (**Fig 3A**). The frequencies of the different genotypes in the Smad3 Fancd2-129B6F2 breeding at day E12.5 recapitulated the predicted normal Mendelian frequency (**Figs 3B and S3A**). The expected Mendelian ratio for the *Smad3*^*-/-*^*Fancd2*^*-/-*^ DKO mice was preserved at this early embryonic stage of development, especially for the Smad3 Fancd2-129B6F2 breeding (**Figs 3C and S3B)**. However, no DKO embryos were observed at day E14.5 (**S3C Fig**), further demonstrating that *Smad3* and the canonical TGFβ pathway are essential for the survival of FA embryos from day E12.5 onwards to birth. Of note, at day E14.5 of gestation, *Fancd2*^*-/-*^ embryos already displayed defective hematopoiesis, as determined by reduced hematopoietic colony numbers *in vitro*, based on a CFU assay of cells derived from mouse fetal liver (**S3D Fig**).

In order to explore the relevance of the canonical TGFβ-*Smad3* pathway during FA ontogenesis, we used targeted RNA sequencing and transcriptional profiling of the TGFβ pathway in *Fancd2*^*-/-*^ embryos **(Fig 3D, *left***). Whole embryos were collected at day E12.5, and the isolated total body RNA was evaluated. As predicted, the mRNAs encoding several components of the TGFβ superfamily were upregulated in *Fancd2*^*-/-*^ embryos, compared to WT embryo controls. These upregulated mRNA transcripts included the *Tgfβ1* and *Tgfβ3* mRNAs, encoding ligands at the apex of the TGFβ pathway. The mRNAs encoding the receptors for these ligands, *Tgfbr2* and *Tgfbr3*, were also upregulated in the *Fancd2*-deficient embryos. This pattern of transcriptional upregulation further suggests a requirement of TGFβ pathway signaling during the embryogenesis of the *Fancd2*^*-/-*^ mice. Of note, mRNAs with opposing functions were also upregulated (**Fig 3D**), including *Smad7*, known as an Inhibitory-Smads, and *Myc*, a proto-oncogene that stimulates cell proliferation and overcomes TGFβ growth suppression [6]. Taken together, these data suggest that compensatory negative feedback mechanisms, counteracting the hyperactive TGFβ pathway, are also present at the organismal level during development of embryos with an FA pathway deficiency.

Overexpression of NHEJ genes, has also been observed in FA cells, and several reports have demonstrated that the canonical TGFβ pathway upregulates these pathways [7, 10, 22]. Since FA is a DNA repair deficiency syndrome, we used targeted RNA sequencing to evaluate the expression of DNA repair genes at day E12.5 of embryonic development (**Fig 3D, *right***). Interestingly, *Trp53* and *Cdkn1a*, which encodes p21, are overexpressed in the whole embryo, confirming that DNA stress responses leading to cell cycle arrest and apoptosis are highly active during development of an FA embryo. Previous studies had indicated that expression of these genes is upregulated in the hematopoietic compartment of human FA embryos [5]. Among the upregulated error-prone DNA repair genes in FA embryos were *Prkdc*, *Mad2l2*, *Trp53bp1*, *Parp1* and *Lig3* (**Fig 3D**). Importantly, overexpression of low-fidelity NHEJ and alternative error-prone genes has detrimental consequences for the FA adult hematopoiesis. However, the access to high-capacity but low-fidelity DNA repair pathways during rapid cell proliferation stages, such as embryogenesis, appears to be critical for ensuring the survival of an embryo with a deficiency in the FA pathway. We speculate that, although toxic for adult hematopoiesis, the activation of these alternative DNA repair pathways must be essential for viable gestation of FA embryos. The expression of TGFβ genes and DNA repair genes was also assessed in *Smad3*^*-/-*^ and Smad3 Fancd2-129B6F2 embryos at day E12.5 (**S3E Fig**). In comparison to wild type, the *Smad3*^*-/-*^ embryos exhibited increased expression of the TGFβ pathway genes *Tgfb1*, *Myc*, and *Bmp3* and increased expression of the DNA repair genes *Mad2l2* and *Trip13*. In comparison to wild type the Smad3 Fancd2-129B6F2 embryos continue overexpressing member of the TGFβ pathway, including the *Tgfb1* ligand. Finally, in comparison to the *Fancd2*^*-/-*^, Smad3 Fancd2-129B6F2 embryos display downregulation of multiple DNA repair genes.

### Activation of the non-canonical ERK pathway in a subset of Smad3 Fancd2-129B6F2 embryos

Extensive literature has shown that cells that lose the canonical TGFβ signaling pathway can retain responsiveness to TGFβ ligands and in some cases become dependent of non-canonical TGFβ pathways (Finnson et al., 2020). We found in our gene expression analysis, that Smad3 Fancd2-129B6F2 embryos overexpress the *Tgfb1* ligand (**S3E Fig middle panel**), and found also that Smad3 Fancd2-129B6F2 adults have increased levels of *Tgfb* ligands in peripheral blood plasma (**S4A Fig**). These results suggest that the TGFβ ligands are not lost in our DKO mice and could signal intracellularly through non-canonical TGFβ pathways, and contribute to retention of the FA phenotype. We therefore hypothesized that the Smad3 Fancd2-129B6F2 embryos surviving to the adult stage might activate intracellular mediators of the non-canonical TGFβ pathway, such as the ERK1 pathway. This non-canonical pathway might in turn stimulate the expression of essential genes in the NHEJ pathway in cells deficient in Smad3. This survival pathway would maintain a hyperactive TGFβ-NHEJ axis in the surviving Smad3 Fancd2-129B6F2 mice and could account for their subsequent defect in hematopoietic improvement.

**Fig 4:**
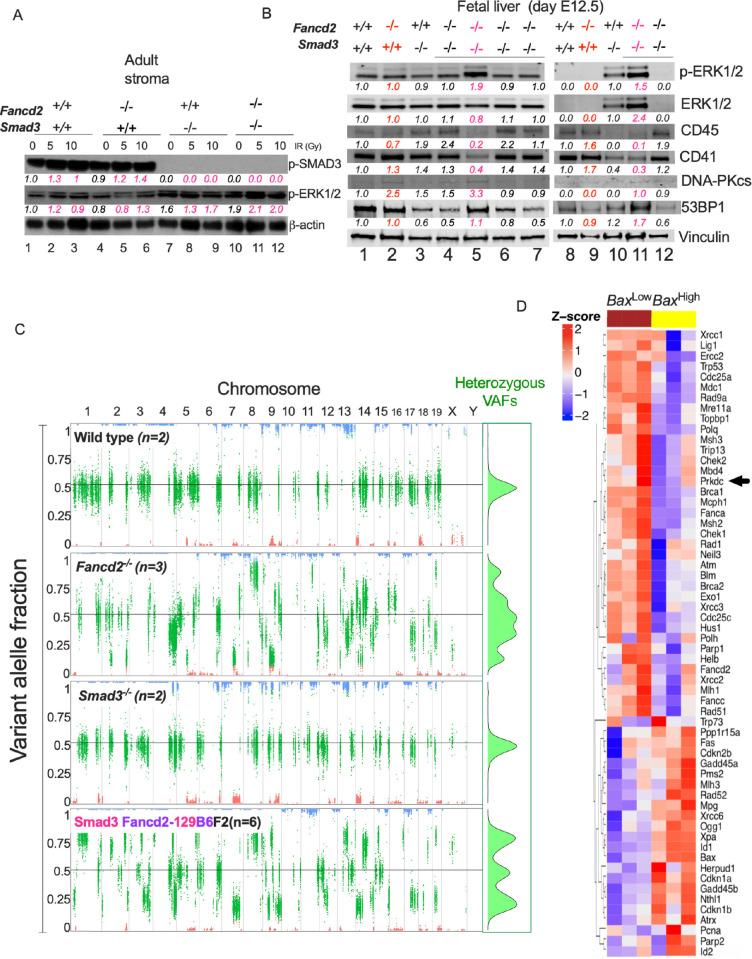
Smad3 Fancd2-129B6F2 embryos with ERK pathway activity express NHEJ components. **(A)** Western blot of the lysates from adult bone marrow stroma cells of mice showing that irradiation activates the canonical phosphorylation of Smad3 in bone marrow stroma of WT and *Fancd2*^*-/-*^ mice and activates the non-canonical ERK1/2 phosphorylation in *Smad3*^*-/-*^ and DKO mice stroma. Quantifications relative to wild type in basal conditions and the loading control are shown below every lane. Pink indicates the irradiated cultures per cell line. **(B)** Western blot of the lysates from E12.5 fetal liver showing that a subset of Smad3 Fancd2-129B6F2 embryos have higher levels of the phospho-ERK protein, suggesting activation of the non-canonical TGFβ pathway. High phospho-ERK embryos have a reduced expression of CD45 and CD41 (CD45 and CD41 were used as surrogates for indication of active hematopoiesis in the fetal liver). High phospho-ERK embryos have higher expression of NHEJ related proteins, DNA-PK and 53BP1. Two western blots are depicted showing a total of two wild type fetal livers, two *Fancd2*^*-/-*^ fetal livers, two *Smad3*^*-/-*^ fetal livers, and six Smad3 Fancd2-129B6F2 fetal livers. Quantifications relative to wild type in basal conditions and the loading control per gel are shown below every lane. Red indicates *Fancd2*^*-/-*^ fetal livers, pink indicates Smad3 Fancd2-129B6F2 fetal livers with high phospho-ERK levels. **(C) Left.** Representative scatter plots of variant allele fractions (VAFs) of the 129/Sv germline variants across the genomes of the analyzed embryos (n = 2 wild type, n = 3 *Fancd2*^*-/-*^, n = 2 *Smad3*^*-/-*^, n = 6 Smad3 Fancd2-129B6F2). Long chromosomal segments with known strain origins are clearly observed. Homozygous reference (C57BL/6) segments are those with VAFs around zero (red); homozygous 129/Sv segments are those with VAFs around one (blue); and heterozygous segments of both strains are those with VAFs between zero and one (green). Wild type and *Smad3*^*-/-*^ embryos show a balanced heterozygosity with VAFs of heterozygous sites clustering around 0.5. *Fancd2*^*-/-*^ and Smad3 Fancd2-129B6F2 embryos show pronounced genomic instability with VAFs of heterozygous sites deviating markedly from 0.5. **Right**. Green inset summarizes the density of VAFs in the heterozygous 129/Sv germline. **(D)** Heatmap for gene expression showing that two types of E12.5 Smad3 Fancd2-129B6F2 embryos exist. Half of the studied E12.5 Smad3 Fancd2-129B6F2 embryos have a *Bax*^High^ gene expression profile (suggesting lethality) and the other half have a *Bax*^Low^ gene expression profile (suggesting survivorship). When profiled for DNA repair related genes, these two groups of E12.5 Smad3 Fancd2-129B6F2 embryos seem to have mutually exclusive gene expression profiles, with down-regulation of alternative error-prone and low fidelity DNA repair genes in the *Bax*^High^ embryos, an effect similar to what has been previously ascribed to pharmaceutical inhibition of the TGFβ pathway. Black arrowhead indicates *Prkdc*, the gene codifying for DNA-PKcs. Data in (C) are represented as variant allele frequency (VAF). Data in (D) are represented as heatmap. See also S4 Fig.

Irradiation is known to activate the TGFβ pathway [16]. We therefore irradiated stromal cells from the different DKO backgrounds and measured phosphorylation of Smad3 (p-Smad3). WT and *Fancd2*^*-/-*^ cell lines upregulated p-Smad3, while the *Smad3*^*-/-*^ and Smad3 Fancd2-129B6F2 cell lines did not. However, phosphorylation of ERK (p-ERK), an alternative signaling mediator of the TGFβ pathway, was observed in *Smad3*^*-/-*^ and Smad3 Fancd2-129B6F2 mice, suggesting that activation of the non-canonical TGFβ-ERK pathway occurs in the surviving Smad3 Fancd2-129B6F2 mice and might contribute to their persistent bone marrow dysfunction (**Figs 4A and S4B**). Inactivation of the SMAD3 pathway was further confirmed by testing SNAIL, a SMAD3 target, that as expected, was reduced in the *Smad3*^*-/-*^ mutant cell line [23]. Interestingly, in the DKO cell line, SNAIL is present, we speculate this might be due to regulation of SNAIL by other transcription factor in the surviving DKO context (**S4C Fig**). A similar response to irradiation was observed in the Smad3 Fancd2-129129F2 stroma (**S4D Fig**), the Smad3 Fancd2-B6B6F2 stroma (**S4E Fig**) and the Smad3 Fancd2-B6129F2 stroma (**S4F Fig**).

Western blot analysis using protein lysates from E12.5 fetal livers confirmed that a subset of Smad3 Fancd2-129B6F2 embryos have high levels of phospho-ERK1/2 (**Fig 4B).** As a surrogate for hematopoietic activity in the fetal liver, we used the CD45 and CD41 markers and found that fetal livers with high phospho-ERK1/2 levels have reduced fetal hematopoiesis and have increased levels of DNA-PKcs and 53BP1, classic NHEJ proteins (**Fig 4B**).

DNA damage has been shown to leave a scar in the genome of DNA repair deficient cell lines [24], we therefore hypothesized that *Fancd2*^-/-^ embryos would have an enhanced genomic instability and tested the same condition in the Smad3 Fancd2-129B6F2 embryos. DNA was extracted from E12.5 embryos and subjected to whole exome sequencing followed by detection of variants. Germline variants were identified, and scatter plots of variant allele fractions (VAFs) (**Fig 4C**), as well as plots of copy number variants (CNVs) (**S4G Fig**) were constructed. The wild type and *Smad3*^*-/-*^ embryos show a balanced heterozygosity with VAFs of heterozygous sites clustering around 0.5, whereas the *Fancd2*^*-/-*^ and Smad3 Fancd2-129B6F2 embryos show pronounced genomic instability with VAFs of heterozygous sites deviating markedly from 0.5 (**S4C**). The CNV analysis showed DNA segments with normal copy ratios close to 1 in wild type embryos, whereas the other genotypes display DNA segments with copy ratios markedly different from 1, especially Smad3 Fancd2-129B6F2, indicating high degree of aneuploidy (**S4G Fig**). This data shows that the deficiency of FA pathway incites genomic instability, which starts early *in utero* in FA embryos and depletion of *Smad3* failed to correct this imbalance.

Previous studies have shown that the TGFβ pathway positively regulates the expression of NHEJ genes [10, 16]. We therefore hypothesized that loss of NHEJ activity would occur in embryos with *Smad3* knockout. Interestingly, when we explored in depth the gene expression profile of Smad3 Fancd2-129B6F2 embryos at day E12.5, we identified two types of Smad3 Fancd2-129B6F2 embryos with opposing gene expression patterns (**Fig 4D**). Approximately half of the embryos overexpressed the pro-apoptotic *Bax* gene (*Bax*^*High*^ Smad3 Fancd2-129B6F2), indicating a survival disadvantage. The other half of embryos downregulated *Bax* (*Bax*^*Low*^ Smad3 Fancd2-129B6F2) and had a presumed survival improvement, based on the number of surviving Smad3 Fancd2-129B6F2 newborns (See **Figs 2B** and **3B**). Of note, the *Bax*^*High*^ Smad3 Fancd2-129B6F2 embryos downregulated the low-fidelity DNA repair pathway genes, such as *Prkdc* that codes for DNA-PKcs, which were otherwise upregulated in *Fancd2*^*-/-*^ embryos (**Fig 3D**). This result was expected and was consistent with previous studies showing downregulation of NHEJ pathway genes following TGFβ pathway inhibition in FA cells [10]. The *Bax*^*Low*^ Smad3 Fancd2-129B6F2 embryos had the opposite gene expression profile. Interestingly, these *Bax*^*Low*^ Smad3 Fancd2-129B6F2 embryos expressed high levels of NHEJ mRNAs, such as *Prkdc*, despite the absence of the canonical Smad3-driven TGFβ pathway (**Fig 4D**).

As we showed that the non-canonical ERK pathway is activated in a subset of Smad3 Fancd2-129B6F2 embryos and in the surviving Smad3 Fancd2-129B6F2 mice, we explored the presence of TGFβ ligands in the supernatant from our LTBMC long-term cultures using an ELISA assay. In this assay we discovered that Smad3 Fancd2-129B6F2 cultures secrete higher levels of TGFβ ligand than the other genotypes (**S4D Fig**), suggesting that TGFβ ligand production is not lost in the surviving Smad3 Fancd2-129B6F2 mice and is being transduced through the ERK non-canonical pathway.

Taken together, these findings suggest that knockdown of *Smad3* in a FA background reduces NHEJ activity and promotes hematopoietic activity; however, the NHEJ pathway is still critical for the survival of FA embryos. Accordingly, the DKO embryos survive by activating an alternative non-canonical TGFβ signaling pathway, through the ERK kinase, in order to upregulate NHEJ.

### Inhibition of the non-canonical TGFβ pathway rescues Smad3 Fancd2-129B6F2 cells from genotoxicity

**Fig 5:**
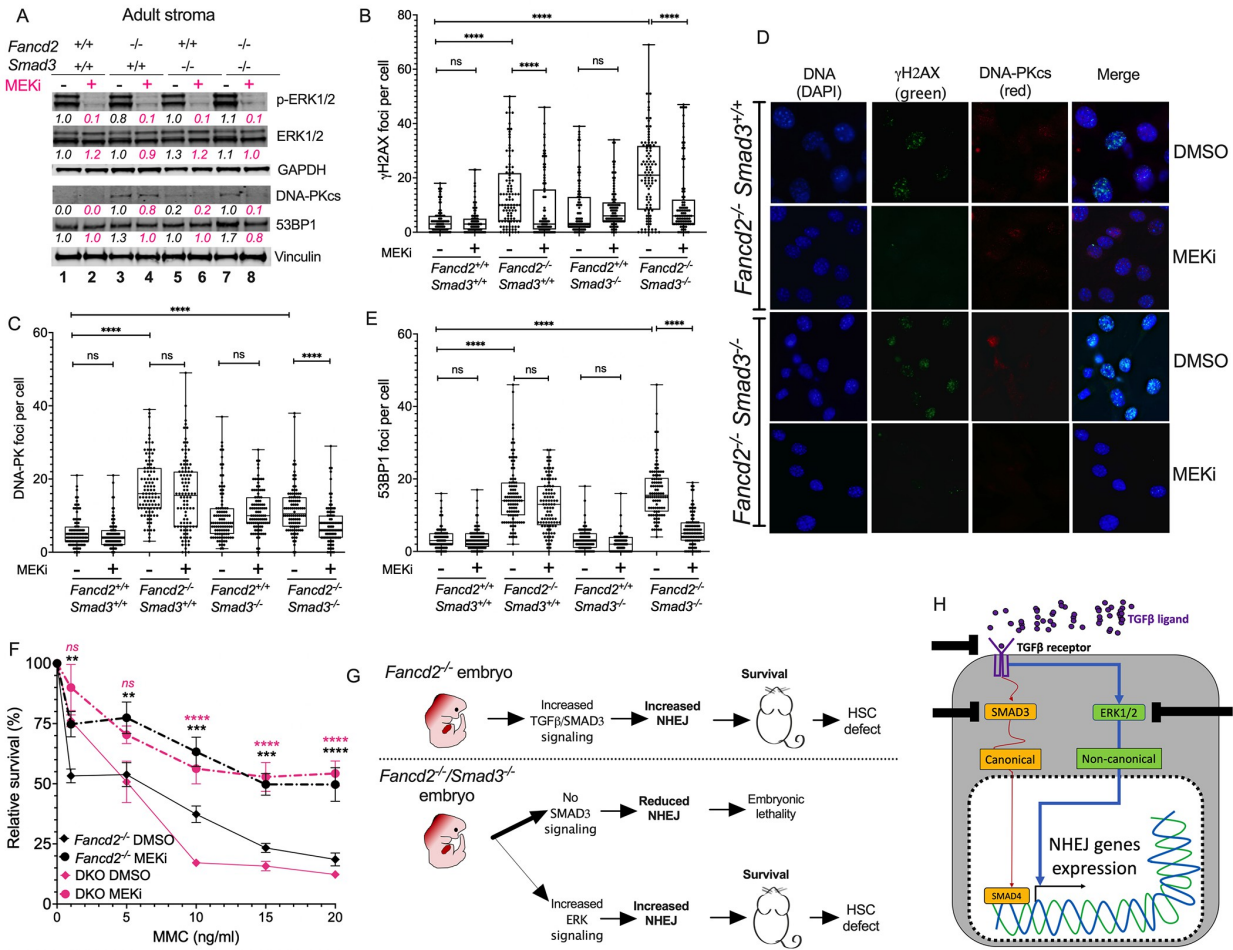
Inhibition of ERK in surviving Smad3 Fancd2-129B6F2 mice stroma reduces NHEJ proteins and improves DNA repair. **(A)** Western blots of the lysates from bone marrow stromal cells showing that inhibition of ERK phosphorylation with the MEK inhibitor PD0325901 reduces the levels of DNA-PKcs in DKO cells but not in *Fancd2*^*-/-*^ cells. A representative blot from three independent experiments is shown. Quantification relative to wild type in basal conditions and the loading control is shown below every lane. Pink indicates samples treated with MEKi PD0325901. **(B)** Quantitation of γH2AX foci by immunofluorescence showing that the MEK inhibitor PD0325901 reduces the number of γH2AX foci in *Fancd2*^*-/-*^ cells and DKO cells. **(C)** Quantitation of DNA-PKcs foci by immunofluorescence showing that the MEK inhibitor PD0325901 significantly reduces the number of DNA-PKcs foci in DKO cells. **(D)** Representative images of γH2AX foci and DNA-PKcs foci in *Fancd2*^*-/-*^ and DKO stromal cells after exposure with MEK inhibitor PD0325901. **(E)** Quantitation of 53BP1 foci by immunofluorescence showing that the MEK inhibitor PD0325901 significantly reduces the number of 53BP1 foci in DKO cells. **(F)** Survival curves of bone marrow stromal cells showing that the MEK inhibitor PD0325901 rescues *Fancd2*^*-/-*^ and Smad3 Fancd2-129B6F2 stromal cells from MMC toxicity. Response of the WT and *Smad3*^*-/-*^ cell lines is shown in S5D Fig. **(G)** Working model. *Upper panel*. FA mouse embryos depend on the canonical TGFβ-SMAD3 pathway for activating the expression of DNA-PKcs and perform NHEJ thus allowing *Fancd2*^*-/-*^ embryo survival in absence of a functional FA pathway. *Lower panel*. Absence of the FA pathway and the canonical SMAD3 pathway is embryonic lethal, however a small fraction of Smad3 Fancd2-129B6F2 embryos activate the non-canonical ERK pathway thus circumventing embryonic death. In both cases surviving mice present with defective hematopoietic stem cells in the bone marrow. **(H)** Schematics showing that the TGFβ ligands and TGFβ receptors can engage the canonical (SMAD3) or the non-canonical (ERK1/2) pathways and activate expression of NHEJ genes. Upon inhibition of the canonical TGFβ pathway, activation of the non-canonical ERK pathway occurs. This possess the potential emergence of resistance to TGFβ pathway inhibitors or refractory to treatment patients, therefore combining TGFβ pathway inhibitors, to avoid the emergence of resistance, has to be considered for designment of future clinical trials. Data in (B), (C) and (E) are represented as boxplots. Data in (F) are represented as mean ± SEM. p values of 0.01 to 0.05 were considered significant (*), p values of 0.001 to 0.01 were considered very significant (**) and p values of < 0.001 were considered extremely significant (***, ****). See also S5 Fig.

We next reasoned that inhibition of the non-canonical ERK pathway in the Smad3 Fancd2-129B6F2 stromal cells derived from adult Smad3 Fancd2-129B6F2 mice would block NHEJ activity. Indeed, inhibition of the non-canonical ERK pathway with the MEK inhibitor (MEKi) PD0325901 resulted in a reduction of DNA-PKcs and 53BP1 protein levels. Interestingly, DNA-PKcs reduction was exclusively observed in the Smad3 Fancd2-129B6F2 cells but not in the *Fancd2*^*-/-*^ cells, suggesting that DNA-PKcs in *Fancd2*^*-/-*^ cells is not under the control of ERK and the non-canonical pathway (**Fig 5A**).

Additional validation of these findings with immunofluorescence microscopy revealed a marked reduction of γH2AX foci in *Fancd2*^*-/-*^ and Smad3 Fancd2-129B6F2 cells exposed to the MEKi (**Fig 5B**) and a reduced number of DNA-PKcs foci (**Fig 5C)** and **Fig 5D**) and 53BP1 foci (**Fig 5E**), especially in Smad3 Fancd2-129B6F2 cells.

The MEKi rescued the *Fancd2*^*-/-*^ and Smad3 Fancd2-129B6F2 cells from the toxicity induced by MMC (**Fig 5F)**. Finally, treatment with LSN3301240, an upstream inhibitor of the TGFβ pathway which blocks TGFβ RI, resulted in a reduction of DNA-PKcs levels in both *Fancd2*^*-/-*^ and Smad3 Fancd2-129B6F2 cell lines, confirming that the TGFβ pathway is active in both cell lines (**S4D and S5B Figs**).

We conclude that during embryogenesis, *Fancd2*^*-/-*^ embryos activate the canonical TGFβ-SMAD3 signaling pathway and thereby upregulate the expression of NHEJ genes, allowing survival in the absence of a functional FA pathway. This NHEJ upregulation is detrimental to adult hematopoiesis later in life (**Fig 5G, upper panel**). On the other hand, if FA embryos lose the canonical TGFβ pathway, due to *Smad3* inactivation, the viable Smad3 Fancd2-129B6F2 embryos will activate alternative components of the TGFβ superfamily for survival. These rare surviving embryos are the exceptions to the synthetic embryonic lethality that prevails upon simultaneous loss of *Fancd2* and *Smad3* (**Fig 5G, lower panel**).

## Discussion

Hyperactivation of the TGFβ pathway is one of multiple abnormalities observed in the bone marrow of patients with FA. This activity impairs the growth of the FA HSPC pool [10]. Accordingly, inhibition of the TGFβ pathway with multiple pharmacological compounds has a positive impact on the proliferation of FA HSPCs [7]. Currently, TGFβ pathway inhibitors are a potential therapeutic option for FA patients who are developing bone marrow failure.

The TGFβ superfamily of ligands is composed of a large number of molecules that modulate multiple processes in a context-dependent manner [11, 25]. The role of the TGFβ pathway as a master regulator during embryogenesis has long been recognized. Some ligands of the TGFβ superfamily are active during early embryogenesis, including Nodal and GDF3, whereas other ligands are critical for later development, organogenesis, and tissue homeostasis, such as TGFβ, activin, myostatin, and other GDFs [13, 26–28].

In recent years, a role for the TGFβ pathway as a modulator of DNA repair has emerged. The TGFβ ligands and extracellular pathway signalers, can respond to DNA damage and modulate the DNA repair pathway choice when DNA double strand breaks are encountered [17, 18]. The SMAD proteins are critical intracellular transducers of TGFβ and have been shown to coordinate the expression of genes involved in DNA repair [10]. Specifically, SMAD proteins upregulate NHEJ gene expression and downregulate HR gene expression. In this work we discovered that both processes modulated by TGFβ converge in FA and further support the contextual versatility of the TGFβ–SMAD signaling pathway.

We previously reported in the context of FA that the overactivity of NHEJ genes is controlled by the TGFβ pathway and that NHEJ, a low-fidelity DNA repair pathway, becomes activated when the FA pathway is disrupted [10]. Importantly, during the S/G2 phase a functional FA pathway inhibits NHEJ activity, thus avoiding the accumulation of gross chromosomal aberrations.

Importantly, we have proven in this and in previous work [7], that pharmacological inhibition of the TGFβ pathway *in vitro* and *in vivo* with AVID200, Galunisertib, and LSN3301240 inhibits the activation of the NHEJ pathway genes (for example, *Lig4* and *Prkdc*), activates the expression of HR genes (for example, *Brca2* and *Xrcc1*), and reduces the DNA damage burden in FA HSPCs [7, 10]. Genetic abrogation of *Smad3* in FA cell lines also leads to an inhibition of the NHEJ pathway and improves their repair capacity and survival. Although, we cannot rule out that HR is upregulated when HSCs enter the cell cycle, thereby resulting in repair of double strand breaks, as previously shown [29, 30], to confirm that TGFβ pathway inhibition creates an HR-competent state, our group has previously used pharmacological inhibitors that interfere with both HR (RAD51 foci formation by B02 inhibitor) and the TGFβ pathway (1D11) in FA cells. Treatment with B02 resulted in reduction of RAD51 foci formation and inhibition of DR-GFP plasmid recombination in a reporter assay. Importantly, 1D11 treatment did not protect FA HSPCs from acetaldehyde-induced genotoxicity when HR was inhibited by B02 [10]. These data showed that TGFβ pathway inhibition upregulates HR and downregulates NHEJ in HSPCs of *Fancd2*^*-/-*^ mice and functionally promote their survival, not only on a cell cycle-dependent manner.

With the exception of *Smad3*^*-/-*^ mutant mice, most mouse models with genetic abrogation of the main components of the TGFβ pathway result in embryonic lethality or death shortly after birth [13]. Thus, the generation of *Fancd2*^*-/-*^ *Smad3*^*-/-*^ double knockout mutants provided an interesting opportunity for testing whether constitutive depletion of *Smad3* would rescue FA mice from the negative effects of excessive NHEJ activity.

We confirmed with our genetic models that, in absence of a functional FA pathway, the TGFβ signaling upregulates the expression of NHEJ genes, such as DNAPK and 53BP1. However, genetically removing *Smad3* along with *Fancd2* resulted in high levels of embryonic lethality during the second half of gestation (day 13.5 onwards), implying that the activity of the NHEJ pathway is critical for FA embryo survival. Therefore, the TGFβ pathway, during FA embryogenesis, provides a compensatory mechanism for survival, promoting repair through NHEJ during the periods in development when rapid cell proliferation occurs, however this comes at the cost of genomic instability appearing early during embryonic development of a FA mice (Figs 4C and S4G). Of note, genetic abrogation itself caused a reduction in the number of weaned DKO pups, but also the genetic background of every cross is significant since specific combinations between the 129/Sv and C57BL/6 strains resulted in higher levels of lethality (Figs 2B and 3), underlying the relevance that inbred mouse strains can have in embryonic lethality.

Gene expression profiling of embryos at day E12.5 confirmed the high demand for the TGFβ pathway in FA embryos in comparison to wild type embryos, since numerous TGFβ factors exhibit increased expression. We are aware that mRNA levels do not strictly reflect protein production, stability, and a final repair outcome. however extensive evidence exists that activation of NHEJ genes occurs in FA pathway deficient cells. Therefore, activation of alternative and low fidelity DNA repair pathways in FA embryos might be their bet for survival, and we are the first to report that overexpression of NHEJ components occurs early in the life of a FA mice embryo (Fig 3D) and is probably also occurring in human FA embryos.

Despite the increased embryonic loss of the *Fancd2*^*-/-*^*Smad3*^*-/-*^ genotype, a small fraction of these DKO embryos do survive through full gestation. Contrary to our original expectation, these DKO newborns display a phenotype resembling FA, including NHEJ overexpression, sensitivity to MMC, bone marrow failure, and reduced bone marrow reconstitution capacity (with slight differences in their response due likely to their genetic background). In short, the loss of *Smad3* appeared to have little effect on the FA phenotype. This paradox prompted us to hypothesize that the surviving DKO mice are rescued by activation of a non-canonical TGFβ pathway, resulting in an exception to the synthetic embryonic lethality imposed by simultaneous deficiency of *Fancd2* and *Smad3*. Analysis of the DKO embryos confirmed that a fraction activated the non-canonical ERK pathway, leading to increased levels of NHEJ proteins and survival. Consistent with this interpretation, the DKO embryos without ERK pathway activation did not display increased NHEJ proteins expression.

After confirming in the adult derived DKO cell lines the overexpression of NHEJ proteins, we inhibited the non-canonical ERK pathway using a MEK inhibitor that prevents phosphorylation of ERK. We observed that this inhibitor rescued DKO cells from MMC sensitivity and reduced the levels of γH2AX, DNA-PK and 53BP1 only in DKO mice but not in *Fancd2*^*-/-*^ mice, confirming that expression of the NHEJ proteins is under control of the non-canonical ERK pathway only in the surviving DKO mice. Finally, we showed that DKO long-term cultures secrete high levels of TGFβ ligands and treatment with LSN3301240, which inhibits TGFβ RI, reduced NHEJ protein levels in both *Fancd2*^*-/-*^ and DKO mice, confirming that the extracellular TGFβ ligands control the canonical Smad3 pathway in *Fancd2*^*-/-*^ mice and control the non-canonical ERK pathway in the surviving DKO mice.

Our results represent an example of how similar genomic mechanisms for survival can be detected in both a multilineage mesenchymal stem cell line derived from a surviving adult DKO mouse and during mid-gestational DKO embryos. The adult DKO mouse marrow stromal cell lines allowed us to conclusively show a genetic adaptation or “rewiring” to by-pass the absence of both the FA pathway and the canonical TGFβ signaling. The upregulated non-canonical ERK pathway for TGFβ signaling was linked with upregulation of NHEJ which was required in surviving embryos. Further studies are needed to discover the mechanisms by which a non-canonical TGFβ pathway is activated in DKO embryos, candidate mechanisms fall in the arena of epigenetics, including changes in DNA methylation, changes in chromatin accessibility or emerging enhancer-gene interactions, which however were beyond the scope of the present report.

Taken together, our data demonstrate that hyperactivation of the TGFβ pathway is a costly trade-off during embryogenesis of FA pathway deficient mice, and probably during human FA embryogenesis. On the one hand, activation of the TGFβ-NHEJ axis is required for survival of the FA embryos. On the other hand, this axis is detrimental for adult hematopoiesis but has the potential to be modulated by pharmacological intervention with inhibitors of TGFβ after birth. Strikingly, we discovered that the TGFβ-NHEJ axis is critical for FA embryo survival; accordingly, in absence of the canonical transducer SMAD3, some DKO embryos have the capacity to rewire their intracellular signaling, engage the non-canonical ERK pathway, and transduce the apical signals coming from the extracellular TGFβ ligands. Our results are an example at the organismal level of how extracellular signaling is coordinated with DNA repair pathway choice and embryonic fate.

Finally, our group has shown that inhibiting the TGFβ pathway at multiple levels using inhibitors of the TGFβ ligands, the TGFβ receptors, and TGFβ transducers (such as SMAD3) improves hematopoiesis in FA preclinical models. Importantly, in this work we show that upon inhibition of the canonical TGFβ transducer, activation of non-canonical TGFβ transducers can occur and possess the potential emergence of resistance to TGFβ inhibitors or refractory to treatment patients. Therefore, this work is a timely precedent that usage of combinations of the TGFβ inhibitors, so as to avoid the emergence of resistance, has to be considered for designment of future clinical trials.

### Limitations of study

Our study has limitations. Having studied more than one FA KO mice, such as *Fanca*^*-/-*^ mice, would have been useful to show that our results apply to more than one FA genotype, however the *Fancd2*^*-/-*^ mouse model recapitulates all the hematopoietic phenotypes exhibited by other FA mice [31, 32].

Exploring additional gestational days would give more information on how embryonic development advances in these DNA repair deficient mice, however DKO embryos are no longer observable on day E14.5 and having objective comparisons on the four genotypes of interest (WT, *Smad3*^*-/-*^, *Fancd2*^*-/-*^ and DKO) was not possible for the present report. However, *Fancd2*^*-/-*^ embryos are viable and studying their full gestation will be of interest.

We did not discover a mechanistic connection between the levels of *Bax* and TGFβ pathway activity, however we observed that Smad3 Fancd2-129B6F2 embryos, despite having the same genotype, have distinct gene expression profiles. Interestingly the levels of *Bax* expression correlated with the expression of NHEJ genes, i.e. lower levels of NHEJ correlated with high levels of *Bax*. This correlation made us hypothesize that those embryos with low NHEJ expression will not survive since *Bax* is a p53 target gene that promotes apoptosis [33]. We also believe that those Smad3 Fancd2-129B6F2 embryos that managed to survive were able to rewire their gene expression network and gain NHEJ expression through the non-canonical ERK, as observed in all the Smad3 Fancd2-129B6F2 weaned pups.

## Material and methods

### Ethics statement

All mice at Dana Farber Cancer Institute were housed according to the Institutional IACUC protocols and maintained four per cage, fed standard laboratory chow, and maintained on deionized sterilized water. All experimental procedures were approved by the Animal Care and Use Committee of the Dana Farber Cancer Institute. Mice were euthanized by CO_2_ asphyxiation or by isoflurane overdose followed by cervical dislocation at the experimental time point or when loss of body weight, diarrhea, progressive dermatitis and any condition interfering with eating or drinking appeared. All mice at the University of Pittsburgh were housed at the Hlllman Cancer Center in a AAAA approved animal facility under the supervision of the Division of Laboratory Animal Research. All animal protocols were approved by the University of Pittsburgh Institute of Animal Control and Use Committee. All mice were euthanized by CO_2_ inhalation followed by cervical dislocation at timepoints defined by the IACUC approved protocol or when showing signs of pain or distress such as loss of appetite, lacking of drinking, loss of 20% of their body weight, hunching of back, ruffling of fur or lethargy.

### Mice

*Fancd2*^*-/-*^ (C57BL/6) [32] and *Fancd2*^*-/-*^ (129/Sv) (***23***) mice were bred in an established colony [34–36]. *Smad3*^*-/-*^ (C57BL/6) [37] and *Smad3*^*-/-*^ (129/Sv) [38] mice were bred from heterozygote breeding pairs obtained from Dr. Katherine Flanders, NIH, and Jackson Laboratories (Bar Harbor, Maine) respectively. The mice were genotyped by isolating DNA from tail clippings and performing PCR using primers described in the following paragraphs. Control mouse strains of C57BL/6, 129/Sv and 129/Sv X C57BL/6 F1 mice were maintained in cages at 37°C with water and food offered ad libitum.

*Fancd2*^*-/-*^ mice and *Smad3*^*-/-*^ mice on both C57BL/6 and 129/Sv backgrounds are infertile. To obtain DKO mice, heterozygous *Fancd2*^*+/-*^ and *Smad3*^*+/-*^ mice were bred. Smad3 Fancd2-129B6F2 (*Smad3*^*-/-*^ (129/Sv) *Fancd2*^*-/-*^ (B6)) mice were obtained by breeding *Smad3*^*+/-*^ (129/Sv) mice with *Fancd2*^*+/-*^ (C57BL/6) mice. Smad3 Fancd2-129129F2 mice were obtained by breeding *Smad3*^*+/-*^ (129/Sv) with *Fancd2*^*+/-*^ (129/Sv) mice. Smad3 Fancd2-B6B6F2 mice were obtained by breeding *Smad3*^*+/-*^ (C57BL/6) mice with *Fancd2*^*+/-*^ (C57BL/6) mice. Smad3 Fancd2-B6129F2 mice were obtained by breeding *Smad3*^*+/-*^ (C57BL/6) mice with *Fancd2*^*+/-*^ (129/Sv) mice (See also S2A Fig). The genotypes of newborns were identified by isolating DNA from the tip of the tail of weaned pups and performing PCR using primers specific for the *Smad3* transgene, mutated *Smad3* transgene, and *Fancd2* transgene or mutated *Fancd2* transgene.

Pregnant mice from the Smad3 Fancd2-129B6F2 breeding were sacrificed at day E12.5 and E14.5, the uterine horns removed, and the liver and placenta were dissected from the embryos. Embryos were genotyped by isolating DNA from the tip of the tail. Total RNA was extracted from the whole embryo, the fetal liver was used for protein extraction and western blot, a mixture of embryo and placenta was used for whole exome sequencing.

The primers for genotyping were 129/Sv *Smad3* primers: wild type forward: 5’-TGA GTT TGC CTT CAA CAT GA; common reverse: 5’-CAC TCT GCC CAG TCC AAA G; mutant forward oIMR1100: 5’-GCT ATC AGG ACA TAG CGT TGG; 129/Sv *Fancd2* primers: forward MG968: 5’-TCA GCC TCA CAT GGA GTT TAA CG; Common reverse MG1007: 5’-AAT TCG CCA ATG ACA AGA CGC; mutant forward MG1008: 5’-CAG GGA TGA AAG GGT CTT ACG C; C57BL/6 *Smad3* primers: forward *Smad3*-1: 5′-CCA CTT CAT TGC CAT ATG CCC TG; reverse *Smad3*-2: 5′-CCC GAA CAG TTG GAT TCA CAC A; mutant forward pLoxpneo 5′-CCA GAC TGC CTT GGG AAA AGC; C57BL/6 *Fancd2* Primers: forward OST2c: 5’-CAT GCA TAT AGG AAC CCG AAG G; reverse OST2a: 5’-CAG GAC CTT TGG AGA AGC AG; mutant forward LTR2b: 5’-GGC GTT ACT TAA GCT AGC TTG).

### Cell lines

Bone marrow stromal cell lines were established by trypsin ionization of the adherent layer of long-term bone marrow cultures at week 4 [35, 36]. Stromal cells (mesenchymal stem cells) were grown in McCoy’s medium supplemented with 10% fetal calf serum and antibiotics and passaged weekly. Clonal lines were established by making the stromal cells into single cell suspension and using flow cytometry to sort single cells into individual wells of a 96 well plate. Once the cells have grown to confluence in the 96 well plates, they are expanded and made into a clonal cell line.

### Isolation of Lin^-^ and LT-HSC from mouse

Mice were sacrificed and the bone marrow from tibia and femurs was harvested by gentle flushing with HBSS++ buffer [Hanks balanced salt solution (10-547F, Lonza) + HEPES (BP299-100, Fisher Scientific) + Fetal bovine serum (F2442, Sigma) + penicillin-streptomycin (15140–122, GIBCO)]. A 70 μM filter was used for filtering the samples and obtaining a single cell suspension. Lin^-^ enrichment was performed with lineage negative selection using the lineage cell depletion kit (130-090-858, Miltenyi).

For isolation of LT-HSCs, identified as Lin^-^Sca-1^+^c-kit^+^ CD150^+^CD48^-^, Lin^-^ cells were incubated with a mixture of biotin-labeled lineage antibody cocktail against CD3, CD11b, CD19, B220, Gr-1 and Ter119 (51-09082J, BD Pharmingen), and fluorochrome conjugate antibodies PE-Cy7-Sca (Clone D7, 558162, BD Biosciences), APC-c-kit (Clone ACK2, 135108, BD Biosciences), Pacific Blue-CD150 (Clone TC15-12F12.2, 115924, Biolegend) and APC-Cy7-CD48 (Clone HM48-1, 47-0481-82, e-Bioscience), followed by incubation with streptavidin-PE secondary antibody (554061, BD). LT-HSCs were sorted with a BD FACSAria cell sorter.

### Alkaline comet assay

The alkaline comet assay was performed with the sorted LT-HSCs using the CometAssay kit (4250-050-K, Trevigen). LT-HSCs were mixed with low-melting-temperature agarose, coated on slides, and incubated in lysis solution overnight at 4°C. Next day cells were incubated during 1 h in unwinding NaOH solution and subjected to a current voltage of 12 V during 30 in a NaOH electrophoresis solution. Slides were dehydrated in ethanol solutions, washed, and stained with SYBRGreen (S7567, Invitrogen). Some cells were exposed to 10 Gy of irradiation using a 137Cs radiation source (model RS2000, Rad source) with a dose rate of 1 Gy/min and used as a positive control for DNA damage. Pictures were taken using a Zeiss Imager Z1 fluorescence microscope. Tail-length analysis was performed using the OpenComet plugin in the Image J software.

### Colony forming unit assay

Clonogenic potential of mouse HSPCs was assessed in CFU assays by plating 20,000 Lin^-^ cells per triplicate in Methocult GF M3434 methylcellulose (03444, Stem Cell Technologies) and cultured for 7 days along with the TGFβ inhibitors Galunisertib or LSN3301240 at 200 nM, 500 nM and 5 μM final concentrations. The mouse hematopoietic colonies were scored after 7 days of culture at 37°C and 5% CO_2_. Clonogenic potential of human HSPC was assessed in CFU assays by plating 3000 CD34^+^ cells per triplicate in human methylcellulose MethoCult H4434 Classic (04434, StemCell Technologies). Human colonies were quantified and classified after 14 days of culture at 37°C and 5% CO_2_. Pictures were taken with the STEMvision System (StemCell Technologies).

### Isolation of human CD34^+^ cells and viral transduction

Fresh healthy bone marrow samples were purchased from Lonza (1M-105, Lonza). Whole bone marrow was incubated with ammonium chloride (07800, StemCell Technologies) for 10 min on ice for lysis of red blood cells followed by a wash with PBS until obtaining a pellet of white cells. CD34^+^ cells were enriched using the Miltenyi kit (19056, Miltenyi Biotech) and following the manufacturer’s instructions.

Isolated human CD34^+^ cells were cultured in non-tissue culture treated plates for 36 hours in StemSpan SFEMII medium (09655, Stem Cell Technologies) with 100ng/ml of the following recombinant human cytokines: SCF (300–07, Peprotech), TPO (300–18, Peprotech), Flt3 (300–19, Peprotech) and IL-6 (200–06, Peprotech). Cells were then transferred to non-TC 96-well plates in a viral prep consisting of fresh media supplemented with polybrene (TR-1003-G, Sigma) and a lentivirus producing a shRNA against human *FANCD2*; a MOI of 50 was used for the scrambled shRNA and a MOI of 100 was used for the *FANCD2* shRNAs. Plates were spun down at 2300 rpm for 30 minutes at RT and incubated for 12–16 hours. Selection media with 1 μg/ml puromycin (MIR 5940, MirusBio) was added to cultures 12–24 hours after viral infection. FA-like cells were selected in this puromycin-containing media for 72 hours.

### Continuous bone marrow cultures and quantification of TGFβ in supernatant

The contents of a femur and tibia of 6-8-week-old mice of each genotype were flushed into 40 cm square plastic flasks in Fisher’s medium supplemented with 25% fetal calf serum and 10^−6^ M Hydrocortisone hemi-succinate [35, 36, 39]. Adherent layers were established by 4 weeks, and cultures fed by demi-depopulation of non-adherent cells and replaced with an equal volume of fresh medium. Cultures were maintained in a CO_2_ incubator and assayed weekly for 35 weeks for number of cobblestone islands, production of nonadherent cells, percent confluency, and day 7 and 14 colony formation [39].

On weeks 9 and 10, cell culture media was obtained from the different cultures, and a TGF-B ELISA (Abcam, Cat. Ab119557) was performed.

### Competitive repopulation assay

Competitive repopulation studies were performed for Smad3 Fancd2-129B6F2 mice (129/Sv *Smad3*^*-/-*^ C57BL/6 *Fancd2*^-/-^) and Smad3 Fancd2-129129F2 mice (*Smad3*^*-/-*^ *Fancd2*^*-/-*^ all on 129/Sv background) [40]. Bone marrow from Smad3 Fancd2-129B6F2, 129/Sv *Smad3*^*-/-*^, or C57BL/6 *Fancd2*^*-/-*^ mice were compared to C57BL/6 bone marrow by isolating bone marrow from male and female mice and mixing with bone marrow from C57BL/6 female and male mice, respectively, at ratios ranging from all male bone marrow to all female bone marrow. For Smad3 Fancd2-129129F2 experiments, bone marrow from male and female 129/Sv *Fancd2*^*-/-*^, 129/Sv *Smad3*^*-/-*^, and Smad3 Fancd2-129129F2 mice was mixed with bone marrow from female and male 129/Sv bone marrow, respectively.

The mixtures were injected intravenously (IV) into C57BL/6 (A) or 129/Sv (B) mice, which had received 10 Gy total body irradiation [41]. Bone marrow from 4–6-week-old mice of each genotype, and of each gender, was prepared as single cell suspensions and injected IV at 10^6^ cells per 10 Gy total body irradiated (TBI) mice, (JL Sheperd Model 68 Cesium irradiator, JL Sheperd and Associates) 300 cGy/min, recipient mice opposite gender in ratios of 1:0, 1:10, 2.5:7.5, 1:1, 7.5:2.5, 10:1 or 0:1 [40]. Groups of recipient *wild type* mice received IV injection of fresh bone marrow 24 hrs after TBI. Transplanted recipient mice were maintained for 120 days, and then bone marrow harvested and assayed for the relative contribution of donor bone marrow from each source by quantitation of Y-chromosome specific DNA [40]. At 120 days after bone marrow injections, the mice were sacrificed, bone marrow isolated, DNA extracted, and PCR performed with primers specific for the Y chromosome. The percent of mice negative for the Y chromosome (Percent Negative for Reconstitution) was plotted against the number of male cells injected.

### Marrow stem cell numbers by flow analysis

Triplicate marrow samples from each mouse were analyzed by single cell, 7-color flow analysis of 1 x 10^7^ cells [40]. Bone marrow was made into single suspensions and incubated with the following antibodies to PE-eFluor 610-CD45, FITC-CD3e, FITC-CD5, FITC-CD8a, FITC-B220, FITC-GR-1, FITC-TER119, FITC-CD41, APC-eFluor 780-CD48, PE-Sca-1, PE-Ch7, and APC-CD150 for one hour. The cells were washed three times with PBS and analyzed by flow cytometry for bone marrow stem cells.

### Protein extraction and western blotting

Whole cell lysates were prepared using RIPA cell lysis buffer (9803, Cell Signaling) and 1 mM PMSF (8553s, Cell Signaling). Lin^-^ cells, cells were cultured for 24 h in StemSpan SFEM medium (09600, StemCell Technologies) containing 2% L-glutamine (25030-081GIBCO), 1% penicillin/streptomycin (15140–122, GIBCO), 100 ng/ml SCF (250-03-10UG, Peprotech) and 100 ng/ml TPO (315-14-10UG, Peprotech). Lin^-^ cells were exposed to TGFβ1 (5 ng/ml) or TGFβ3 (5 ng/ml) along with LSN3301240 (5 μM) during 2 h. Western blots were performed using the following antibodies SMAD2/3 (86855, Cell Signalling), phospho-SMAD2 (ab3849, Millipore), phospho-SMAD2/3 (8828s, Cell Signaling) and Vinculin (sc-25336, Santa Cruz) antibodies.

Bone marrow stromal cell lines were exposed to irradiation (5 or 10 Gy), MEK inhibitor PD0325901 (10 μM) or TGFβ inhibitor LSN3301240 (5 μM). Western blots were performed with the following antibodies DNA-PKcs, 53BP1, RAD51, phospho-SMAD3 (9502s, Cell Signaling Technology, Danvers, MA, USA), p-ERK and actin (Santa Cruz Biotechnology Inc., Dallas, TX, USA). Antibodies for analysis of fetal liver were DNA-PKcs, 53BP1, pERK1/2, total ERK1/2, CD45 CD41 and Vinculin.

### DNA damage sensitivity assay

For Mitomycin C sensitivity assay, mitomycin C was added daily to Smad3 Fancd2-129B6F2, Smad3 Fancd2-129129F2, Smad3 Fancd2-B6B6F2 and Smad3 Fancd2-B6129F2 bone marrow stromal cell lines for 3 consecutive days at concentrations of 0, 5, 10, 15 or 20 ng/ml per day. The cells were then trypsinized and prepared as single cell suspensions, plated in each of 4 well plates at 500 cells per well and then incubated at 37°C in a CO_2_ incubator for 7 days. The plates were stained with crystal violet and colonies of greater than 50 cells counted [35, 36]. For IR sensitivity assay, the Smad3 Fancd2-129B6F2 stromal cell line was exposed to 2.5, 5 and 10 Gy of IR, let to recover during 5 days and viability assessed with the CellTiter Glo viability assay.

### RNA extraction and targeted RNA sequencing

RNA was extracted from embryos using the Midi RNA extraction kit (74034, QIAGEN). RNA quality was assessed using an Agilent Bioanalyzer and RNA Nano Chip in the Biopolymers Facility of Harvard Medical School. For gene expression analysis a QIAseq Targeted RNA panel targeted was designed (CRHS-10510Z-219-12, 333022, QIAGEN). Index assignment was done using the QIAseq Targeted RNA 96-index I kit (333117, QIAGEN). Libraries were sequenced in an Illumina NextSeq 500 Mid Output flow cell and run in an Illumina NextSeq 500 sequencer in the Biopolymers Facility of Harvard Medical School.

### Gene expression analysis

Targeted RNAseq was performed using a panel of 203 mouse genes belonging to the DNA repair and TGF-β pathways. The panel with 9 reference genes were designed using the QIAseq Targeted RNA Panel with molecular barcode technology. Libraries were prepared according to manufacturer’s instructions. Next-generation sequencing was performed on the Illumina NextSeq 500 sequencer according to manufacturer’s instructions in the Biopolymers Facility at Harvard Medical School (https://www.genome.med.harvard.edu/).

Targeted RNAseq data were preprocessed as follows. First, QIAGEN’s GeneGlobe online portal was used to quantify gene expression counts from the number of unique molecular barcodes in the raw sequencing reads. Next, suitable reference genes were selected using geNorm [42] to be used for normalization. Finally, the gene expression counts were normalized using the selected reference genes and transformed into log2-counts per million (log2-CPM) values.

The relationship between the samples was examined using the Principal Component Analysis (PCA) plots, and the multi-dimensional scaling (MDS) plots which were generated by the plotMDS function of the limma R package [43]. PCA and MDS plots indicated the library preparation day could be a potential confounding factor.

Differential gene expression analyses were performed using the edgeR package [44], and the DESeq2 package [45], with the raw expression counts and the selected reference genes as inputs, and with library preparation day as a covariate. Both algorithms were used to have more confidence in the results. The estimated log-fold-changes of the two algorithms were confirmed to be highly similar. Multiple testing correction to control the false discovery rate (FDR) was performed using the Benjamini-Hochberg procedure on the p-values. The volcano plots of the edgeR results were generated using the ggplot2 package in R.

### DNA extraction and whole exome sequencing

DNA from day E12.5 embryos and placentas were extracted using the DNA extraction kit from QIAGEN. DNA samples were submitted to GENEWIZ (South Plainfield, NJ) for library preparation and sequencing. Sequencing libraries were prepared with the HiseqX V2.5 sequencing kit and sequenced in a Illumina platform with a sequencing configuration of HiSeq, 2x150bp sequencing, single index. A total data output of ~350M raw paired-end reads were obtained per lane. FASTQ files were obtained for mutational signature extraction.

### Whole exome sequence analysis

The paired-end whole exome sequence data were preprocessed following GATK Best Practices. Briefly, for each sample, the raw paired-end sequencing reads were mapped to the reference mouse genome C57BL/6J (GRCm38; mm10) using the Burrows-Wheeler aligner [46] (BWA-MEM; version 0.7.17). Duplicate reads were marked using Picard (version 2.19). The indels were realigned using GATK3 [47] (version 3.8). Finally, base quality scores were recalibrated using GATK4 (version 4.1).

Variant calling was performed using GATK3 UnifiedGenotyper (version 3.8) in the targeted coding regions. All 13 embryos were called together without down sampling. BCFtools [48] was then used to filter out some potentially low-quality variants with these parameters: “-g3 -G10 -e’QD<2.0 || FS>60.0 || SOR>5.0 || MQ<30’”. Only biallelic SNVs were selected for signature analysis. We discovered a total of over 128k SNVs from all 13 embryos.

We next identified the germline and somatic variants. One large source of known germline variants came from the 129S1 strain. We identified over 57k known 129S1 germline variants within the targeted regions, that were discovered from the whole genome sequencing efforts at The Wellcome Trust Sanger Institute [49]. Over 90% of the known 129S1 germline variants were rediscovered in the exomes of the embryos.

The scatter plots of the VAFs of the 129S1 germline variants were plotted using ggplot2 R package.

To identify other germline variants and other false variants, and somatic variants, we developed a simple procedure: if a variant was present in more than one embryo, it’s likely to be germline or other false variants; and if it was seen in only one embryo it is likely to be somatic. The exact procedure was as follows: if the highest variant allele fraction (VAF) was above 0.05, and the second highest VAF was more than 40% of the highest VAF, then it’s germline or false variant, else it’s a somatic variant. Variants with the highest VAF smaller than 0.05 were excluded. These thresholds were optimally picked so that most of the known 129S1 germline variants were identified. Upon manual visual inspection of VAF plots, we identified and removed several more clusters of variants that were likely germline, they appeared in clusters in only one embryo. Copy number analysis was performed following the GATK (version 4.1) workflow for detection of copy ratio alterations and allelic segments. The analysis-ready BAMs were used as inputs. The two wild type embryos were used to create the panel of normals (PoN) reference.

### Immunofluorescence

Cells were grown on coverslips and fixed for 15 min in 4% paraformaldehyde (30525-89-4, Electron Microscopy Sciences) in PBS, then permeabilized with ice-cold methanol during 2 min at room temperature, washed immediately with PBS and incubated for 1 h in blocking buffer (10% Triton X-100 diluted in PBS with 10% normal goat serum.

Cells were incubated with primary antibodies anti- phospho-histone H2A.X (Ser 139) antibody (2577s, Cell signaling) anti-DNA-PKcs and anti-53BP1 diluted in IF buffer (1% BSA diluted in PBS and 10% Triton X-100 diluted in PBS) in a humidified chamber overnight at 4°C. The next day, cells were washed with PBSs threetimes and incubated for 1 h with secondary antibodies anti rabbit Alexa Fluor-488 (20E3, Cell Signaling) and Alexa Fluor- diluted 1:200 in IF buffer.

Slides were washed three times in PBS, counterstained and mounted with ProLong Gold antifade reagent with DAPI (P36931, Life Technologies). Images were taken with a Zeiss Imager fluorescent microscope.

### Statistical analysis

Densitometry in western blots was analyzed with ImageJ software. Relative expression was calculated based on the intensity of each band over the intensity of its loading control. Data were normalized using WT levels or basal conditions as reference.

For analysis of frequency of births, we tested whether the frequency of births was significantly different from an expected frequency of 1 in 16, using a two-sided proportional test with p-values adjusted for multiple tests using the Bonferroni method. For competitive repopulation assay analysis, a single-hit model was fitted for each cell type, and comparison between the two cell types was done with the likelihood ratio test using the asymptotic chi-square approximation followed by the Bonferroni test.

For in vitro assays, we used the D’Agostino-Pearson, Shapiro-Wilk and Kolmogorov Smirnoff tests when required for assessing normality. We identified outliers with the ROUT method. 2-way ANOVA and Dunn’s multiple comparisons test were used for comparing experimental groups. Two-tailed p values for statistical analysis were obtained using Student’s t test.

p values of 0.01 to 0.05 were considered significant (*), p values of 0.001 to 0.01 were considered very significant (**) and p values of < 0.001 were considered extremely significant (***, ****). Graphpad Prism 8 and the Elda function in statmod package of R were used for statistical analysis.

## Supporting information

S1 Fig **(A)** Galunisertib and LSN3301240 improve the clonogenic capacity of FA-like HSPCs measured in a CFU assay. FA-like human primary bone marrow HSPCs were generated by transducing primary bone marrow CD34+ cells with Lentivirus encoding two different shRNAs against FA gene *FANCD2*. These FA-like cells were then cultured in methylcellulose medium containing Galunisertib or LSN3301240 for 10 days and hematopoietic colonies (CFUs) were counted for assessing clonogenic growth of progenitors. **(B)** Stromal cell lines generated from WT and *Fancd2*^*-/-*^ mice were cultured in the presence of Galunisertib and MMC and survival was determined. Galunisertib did not show an efficient rescue of FA cells from MMC. Data in (A) and (B) are represented as mean ± SEM. p values of 0.01 to 0.05 were considered significant (*), p values of 0.001 to 0.01 were considered very significant (**) and p values of < 0.001 were considered extremely significant (***, ****).
(TIFF)

S2 Fig **(A)** Breeding scheme for *Smad3* and *Fancd2* double knockout (DKO) mice. *Smad3*^*+/-*^ mice were bred with *Fancd2*^*+/-*^ mice to obtain F1 mice which are heterozygous for both *Smad3* and *Fancd2*. To get the DKO mice, the F1 mice which are heterozygous for both genes were bred together to get the mice which are homozygous for *Smad3*^*-/-*^ *Fancd2*^*-/-*^ (DKO). The parental mouse strains for Smad3 Fancd2-129B6F2 mice were 129/Sv *Smad3*^*+/-*^ mice and C57BL/6 *Fancd2*^*+/-*^ mice. The Smad3 Fancd2-129129F2 mice were bred from 129/Sv *Smad3*^*+/-*^ mice and 129/Sv *Fancd2*^*+/-*^ mice. For the Smad3 Fancd2-B6B6F2 mice the parental mice were *Smad3*^*+/-*^ and *Fancd2*^*-/-*^ on a C57BL/6 background. The Smad3 Fancd2-B6129F2 parental strains were 129/Sv *Smad3*^*+/-*^ mice and C57BL/6 *Fancd2*^*+/-*^ mice. **(B)** *In vitro* LTBMC assay showing that adult Smad3 Fancd2-129129F2, Smad3 Fancd2-B6B6F2 and Smad3 Fancd2-B6129F2 mice have a reduced production of bone marrow hematopoietic progenitors, similar to a *Fancd2*^*-/-*^ mouse bone marrow genotype. Results are presented as cumulative day 14 CFU-GEMM forming cells. **(C)** Competitive repopulation capacity of the bone marrow in transplant assays showing that hematopoietic cells derived from adult Smad3 Fancd2-129129F2 mice have a reduced competitive repopulation capacity. Competitive repopulation assays were not possible with the Smad3 Fancd2-B6B6F2 and Smad3 Fancd2-B6129F2 crosses due to reduced weaned pups numbers. **(D)** Survival of bone marrow -derived stromal cell lines in presence of MMC showing that cells from Smad3 Fancd2-129129F2, Smad3 Fancd2-B6B6F2 and Smad3 Fancd2-B6129F2 are hypersensitive to MMC. Data in (A), (B) and (C) are represented as mean ± SEM. p values of 0.01 to 0.05 were considered significant (*), p values of 0.001 to 0.01 were considered very significant (**) and p values of < 0.001 were considered extremely significant (***, ****).
(TIFF)

S3 Fig **(A)** Reduced frequencies of Smad3 Fancd2-129129F2 embryos at day E12.5. **(B)** The frequency of Smad3 Fancd2-129129F2 embryos at day E12.5 is even more compromised than in the Smad3 Fancd2-129B6F2 breeding, thus limiting embryo analysis. **(C)** The expected Mendelian frequency for Smad3 Fancd2-129B6F2 is not observed at day E14.5, suggesting embryo loss. **(D)** Assessment of fetal hematopoiesis capacity at day E14.5 in a CFU assay with embryo livers. *Fancd2*^*-/-*^ embryos have reduced CFU capacity at day E14.5, indicating a hematopoietic defect *in utero*. No Smad3 Fancd2-129B6F2 embryos were available for analysis at day E14.5. **(E)** Differential gene expression analysis during day E12.5 in the different mouse genotypes using a targeted RNAs sequencing panel of TGFβ and DNA repair genes. **Upper panel.** Genes differentially expressed in the *Smad3*^*-/-*^ embryos in comparison to wild type embryos. **Middle panel.** Genes differentially expressed in the Smad3 Fancd2-129B6F2 embryos in comparison to wild type embryos. **Lower panel.** Genes differentially expressed in the Smad3 Fancd2-129B6F2 embryos in comparison to *Fancd2*^*-/-*^ embryos. Data in (A), (B) and (C) are represented as bar plots. Data in (D) are represented as mean ± SEM. Data in (E) are represented as volcano plots. p values of 0.01 to 0.05 were considered significant (*), p values of 0.001 to 0.01 were considered very significant (**) and p values of < 0.001 were considered extremely significant (***, ****).
(TIFF)

S4 Fig **(A)** Quantification of TGFβ levels in the cell culture media obtained from the LTBMC assay. LTBMCs were established from the bone marrow of WT, *Smad3*^*-/-*^*Fancd2*^*+/+*^, *Smad3*^*+/+*^*Fancd2*^*-/-*^ and Smad3 Fancd2-129B6F2 mice. The cell culture media was obtained after weeks 9 and 10 following establishment of the cultures and TGFβ levels were measured using a TGFβ ELISA kit. Plot shows the average of weeks 9 and 10. Smad3 Fancd2-129B6F2 cultures produce more TGFβ ligand than the cultures derived from other genotypes. Data are represented as mean ± SEM. p values of 0.01 to 0.05 were considered significant (*), **(B)** Western blots of the lysates from adult bone marrow stromal cells of Smad3 Fancd2-129B6F2 mice showing total Smad3 levels and total ERK levels after irradiation. Quantifications relative to wild type in basal conditions and the loading control are shown below every lane. Pink indicates the irradiated cultures per cell line. **(C)** Western blots of the lysates from adult bone marrow stromal cells of Smad3 Fancd2-129B6F2 mice showing SNAIL levels after irradiation. Quantifications relative to wild type in basal conditions and the loading control are shown below every lane. Pink indicates the irradiated cultures per cell line. **(D)** Western blots of the lysates from stromal cell lines showing the response of the canonical SMAD3 pathway and the non-canonical ERK pathway in cell lines derived from Smad3 Fancd2-129129F2 mice after irradiation. **(E)** Western blots of the lysates from stromal cell lines showing the response of the canonical SMAD3 pathway and the non-canonical ERK pathway in cell lines derived from Smad3 Fancd2-B6B6F2 mice after irradiation. **(F)** Western blots of the lysates from stroma cell lines showing the response of the canonical SMAD3 pathway and the non-canonical ERK pathway in cell lines derived from Smad3 Fancd2-B6129F2 mice after irradiation. **(G)** Representative plots of copy number variants (CNVs) across the genomes of representative embryos, containing denoised copy ratios with segmentation. Wild type shows segments with normal copy ratios of 1. In contrast, *Fancd2*^*-/-*^ and Smad3 Fancd2-129B6F2 embryos show segments with copy ratios markedly different from 1, especially Smad3 Fancd2-129B6F2, indicating high degree of aneuploidy. These CNV data represent total copy numbers across the genome, whereas the variant allele fraction (VAF) data in Fig 4C represent relative copy numbers of the two homologous chromosomes. Both provide complementary signals of genomic instability (n = 2 wild type, n = 3 *Fancd2*^*-/-*^, n = 2 *Smad3*^*-/-*^, n = 6 Smad3 Fancd2-129B6F2).
(TIFF)

S5 Fig **A)** Survival curves of WT and *Smad3*^*-/-*^ stromal cell lines in presence of MMC and the MEK inhibitor PD0325901. **B)** Western blots of the lysates from stromal cell lines showing that inhibition of TGFβ RI with LSN3301240 reduces the levels of DNA-PKcs in cells from both *Fancd2*^*-/-*^ and Smad3 Fancd2-129B6F2 mice. A representative blot from two independent experiments is shown. Quantification relative to wild type in basal conditions and the loading control is shown below every lane. Pink indicates samples treated with LSN3301240. Data in (A) are represented as mean ± SEM. p values of 0.01 to 0.05 were considered significant (*), p values of 0.001 to 0.01 were considered very significant (**) and p values of < 0.001 were considered extremely significant (***, ****).
(TIFF)

S1 Data All the numerical data used for generating the graphs shown in this manuscript are presented in the dataset. (XLSX)

S1 Table Findings on the DKO (Fancd2-/- Smad3-/-) breeding. This Table summarizes the phenotype observed in our DKO crosses. It can be observed variability in the number of pups and etention of the FA phenotype.
(TIFF)
