# Supplementary figures and images for: TGFβ pathway is required for viable gestation of Fanconi anemia embryos
Source: PLoS Genet. 2022 Nov 28;18(11):e1010459. doi: 10.1371/journal.pgen.1010459 (PMC9731498; doi:10.1371/journal.pgen.1010459)

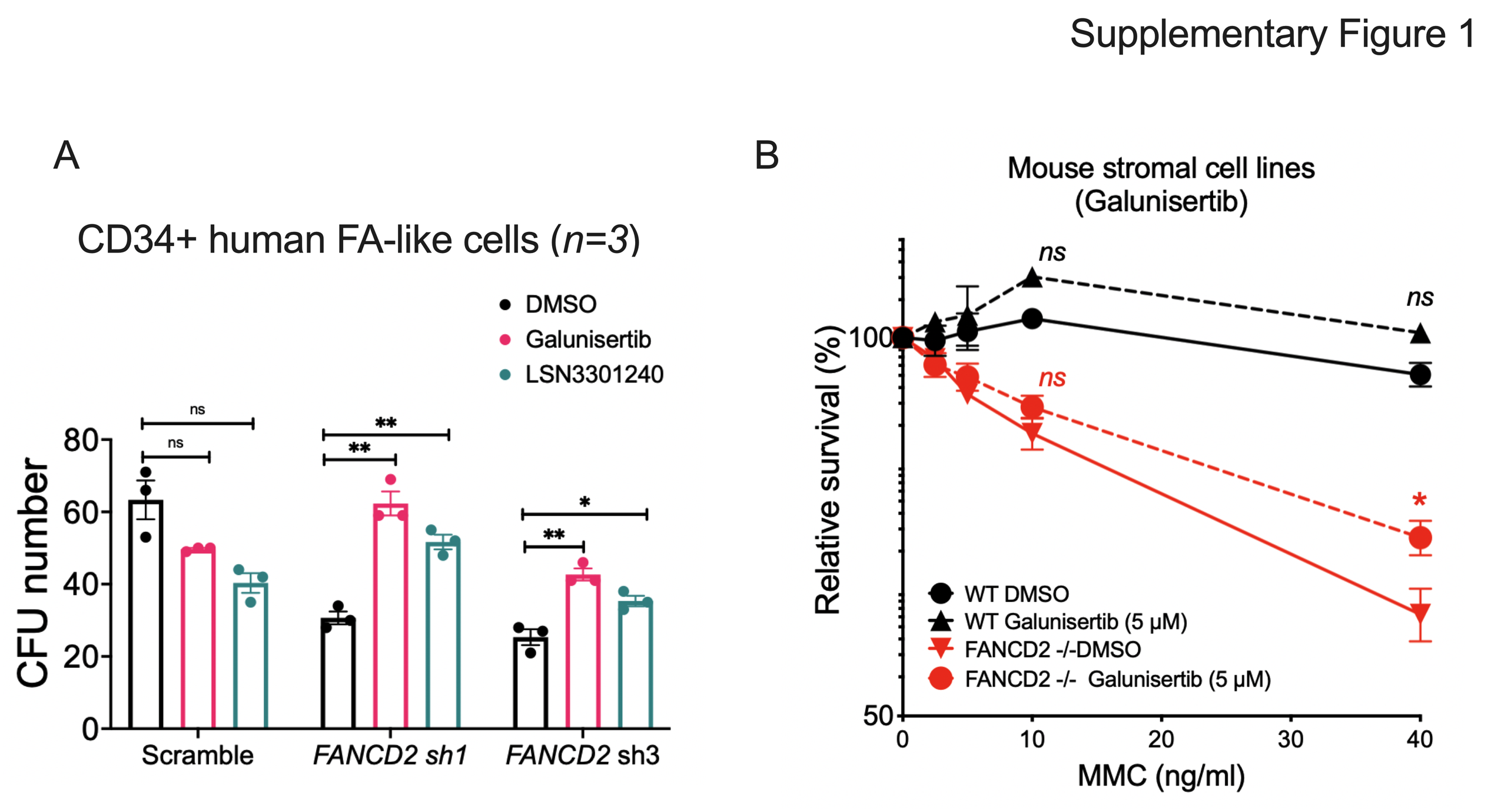

Supplement: S1 Fig — (A) Galunisertib and LSN3301240 improve the clonogenic capacity of FA-like HSPCs measured in a CFU assay. FA-like human primary bone marrow HSPCs were generated by transducing primary bone marrow CD34+ cells with Lentivirus encoding two different shRNAs against FA gene FANCD2. These FA-like cells were then cultured in methylcellulose medium containing Galunisertib or LSN3301240 for 10 days and hematopoietic colonies (CFUs) were counted for assessing clonogenic growth of progenitors. (B) Stromal cell lines generated from WT and Fancd2-/- mice were cultured in the presence of Galunisertib and MMC and survival was determined. Galunisertib did not show an efficient rescue of FA cells from MMC. Data in (A) and (B) are represented as mean ± SEM. p values of 0.01 to 0.05 were considered significant (*), p values of 0.001 to 0.01 were considered very significant (**) and p values of < 0.001 were considered extremely significant (***, ****). (TIFF) [file pgen.1010459.s001.tiff]

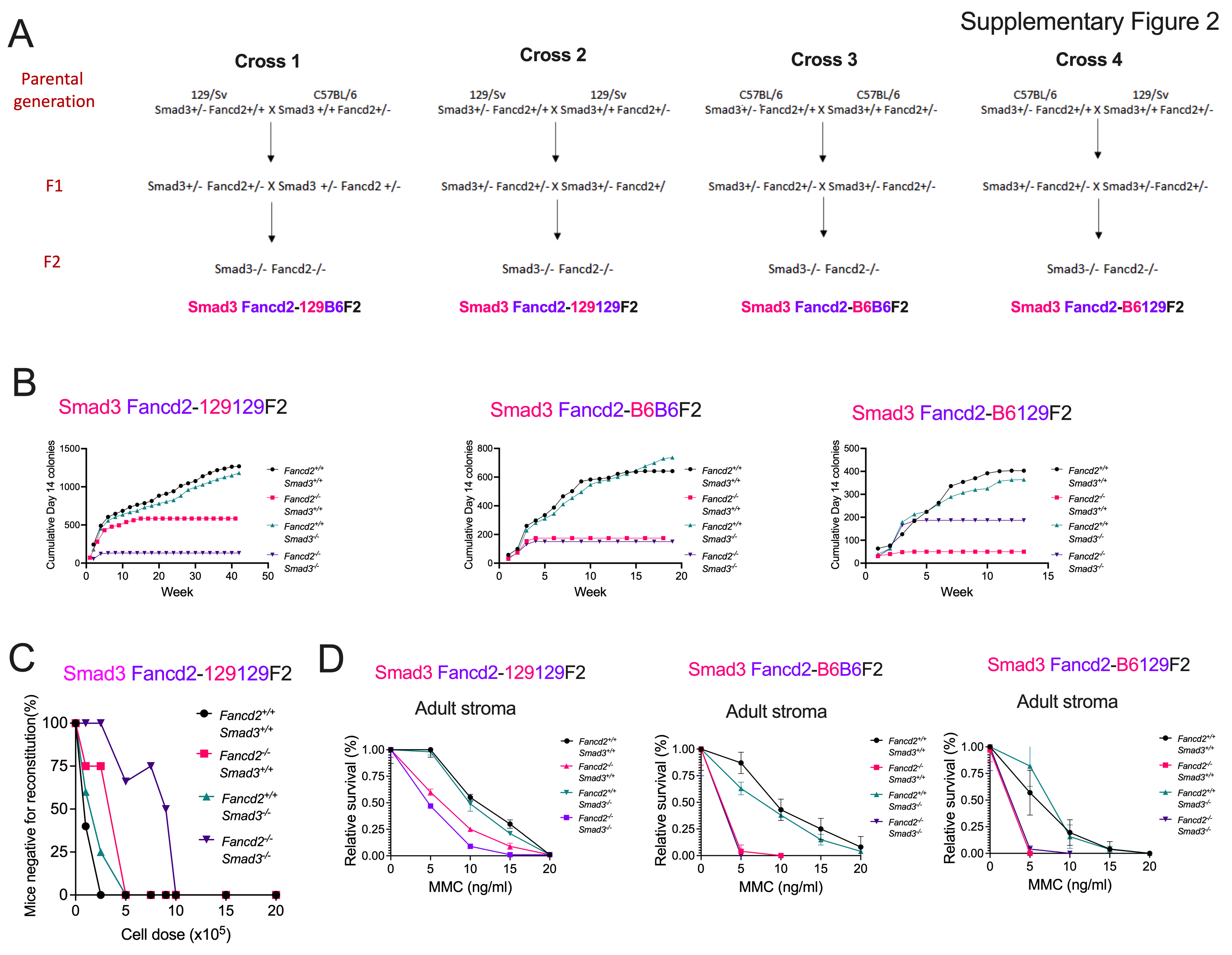

Supplement: S2 Fig — (A) Breeding scheme for Smad3 and Fancd2 double knockout (DKO) mice. Smad3+/- mice were bred with Fancd2+/- mice to obtain F1 mice which are heterozygous for both Smad3 and Fancd2. To get the DKO mice, the F1 mice which are heterozygous for both genes were bred together to get the mice which are homozygous for Smad3-/- Fancd2-/- (DKO). The parental mouse strains for Smad3 Fancd2-129B6F2 mice were 129/Sv Smad3+/- mice and C57BL/6 Fancd2+/- mice. The Smad3 Fancd2-129129F2 mice were bred from 129/Sv Smad3+/- mice and 129/Sv Fancd2+/- mice. For the Smad3 Fancd2-B6B6F2 mice the parental mice were Smad3+/- and Fancd2-/- on a C57BL/6 background. The Smad3 Fancd2-B6129F2 parental strains were 129/Sv Smad3+/- mice and C57BL/6 Fancd2+/- mice. (B) In vitro LTBMC assay showing that adult Smad3 Fancd2-129129F2, Smad3 Fancd2-B6B6F2 and Smad3 Fancd2-B6129F2 mice have a reduced production of bone marrow hematopoietic progenitors, similar to a Fancd2-/- mouse bone marrow genotype. Results are presented as cumulative day 14 CFU-GEMM forming cells. (C) Competitive repopulation capacity of the bone marrow in transplant assays showing that hematopoietic cells derived from adult Smad3 Fancd2-129129F2 mice have a reduced competitive repopulation capacity. Competitive repopulation assays were not possible with the Smad3 Fancd2-B6B6F2 and Smad3 Fancd2-B6129F2 crosses due to reduced weaned pups numbers. (D) Survival of bone marrow -derived stromal cell lines in presence of MMC showing that cells from Smad3 Fancd2-129129F2, Smad3 Fancd2-B6B6F2 and Smad3 Fancd2-B6129F2 are hypersensitive to MMC. Data in (A), (B) and (C) are represented as mean ± SEM. p values of 0.01 to 0.05 were considered significant (*), p values of 0.001 to 0.01 were considered very significant (**) and p values of < 0.001 were considered extremely significant (***, ****). (TIFF) [file pgen.1010459.s002.tiff]

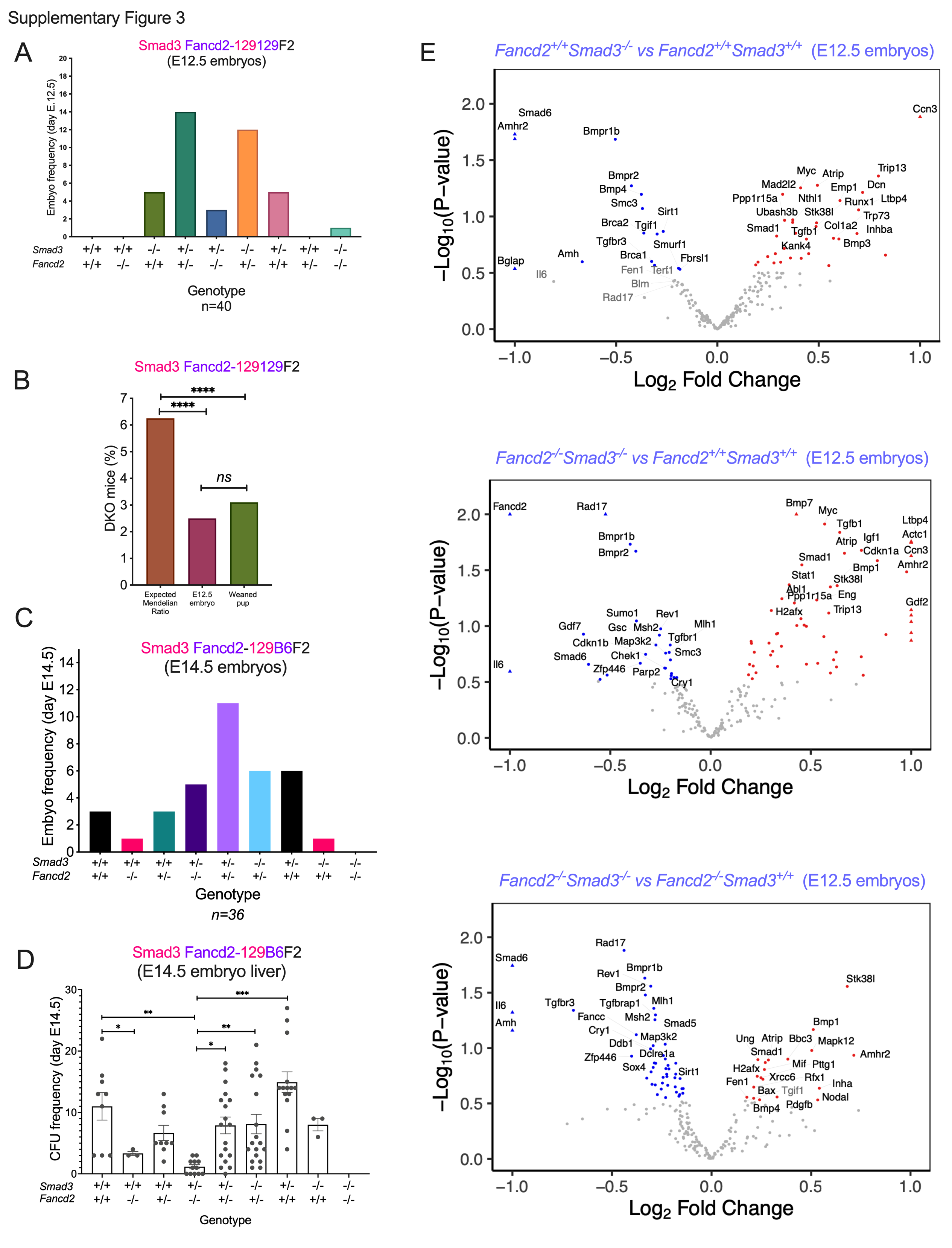

Supplement: S3 Fig — (A) Reduced frequencies of Smad3 Fancd2-129129F2 embryos at day E12.5. (B) The frequency of Smad3 Fancd2-129129F2 embryos at day E12.5 is even more compromised than in the Smad3 Fancd2-129B6F2 breeding, thus limiting embryo analysis. (C) The expected Mendelian frequency for Smad3 Fancd2-129B6F2 is not observed at day E14.5, suggesting embryo loss. (D) Assessment of fetal hematopoiesis capacity at day E14.5 in a CFU assay with embryo livers. Fancd2-/- embryos have reduced CFU capacity at day E14.5, indicating a hematopoietic defect in utero. No Smad3 Fancd2-129B6F2 embryos were available for analysis at day E14.5. (E) Differential gene expression analysis during day E12.5 in the different mouse genotypes using a targeted RNAs sequencing panel of TGFβ and DNA repair genes. Upper panel. Genes differentially expressed in the Smad3-/- embryos in comparison to wild type embryos. Middle panel. Genes differentially expressed in the Smad3 Fancd2-129B6F2 embryos in comparison to wild type embryos. Lower panel. Genes differentially expressed in the Smad3 Fancd2-129B6F2 embryos in comparison to Fancd2-/- embryos. Data in (A), (B) and (C) are represented as bar plots. Data in (D) are represented as mean ± SEM. Data in (E) are represented as volcano plots. p values of 0.01 to 0.05 were considered significant (*), p values of 0.001 to 0.01 were considered very significant (**) and p values of < 0.001 were considered extremely significant (***, ****). (TIFF) [file pgen.1010459.s003.tiff]

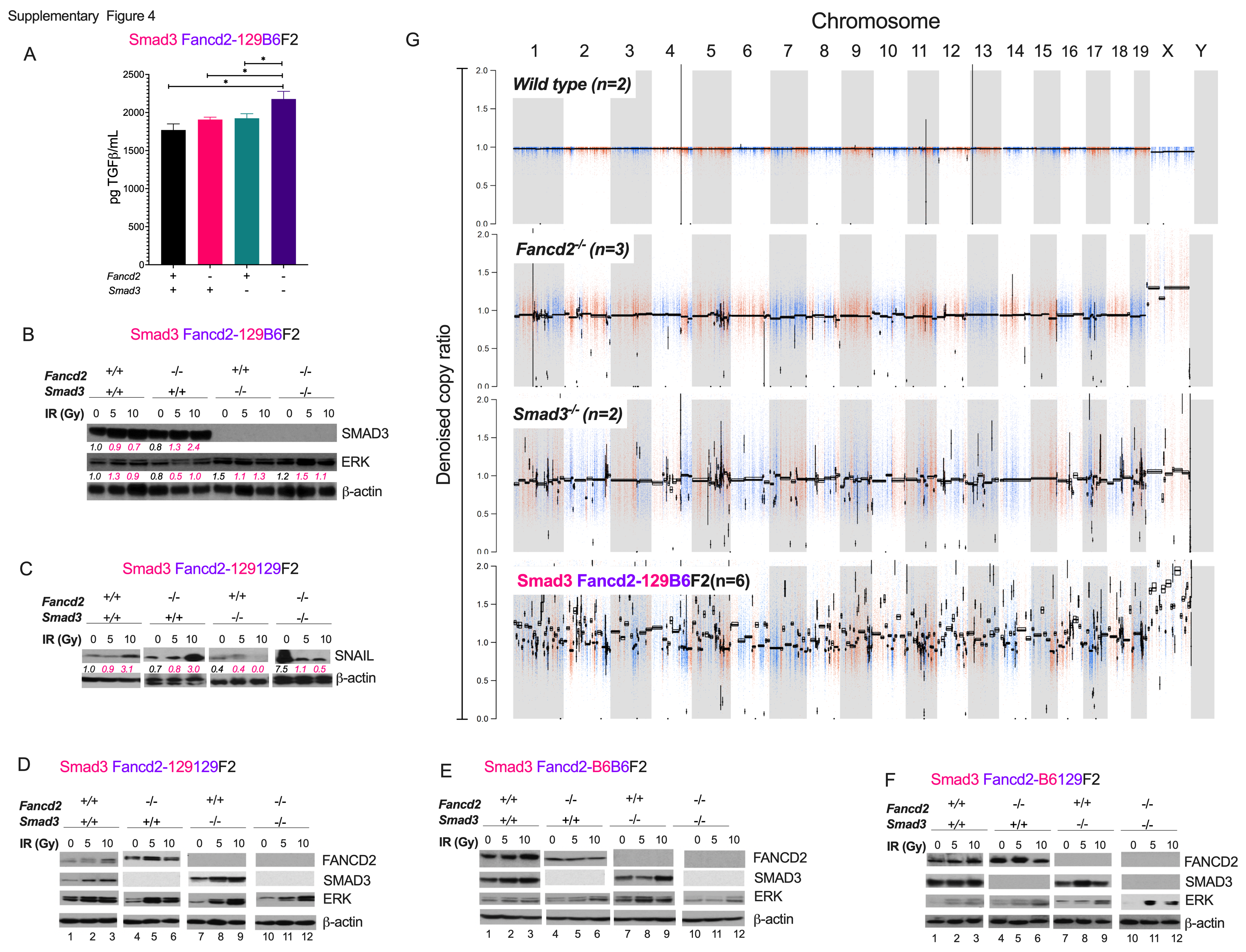

Supplement: S4 Fig — (A) Quantification of TGFβ levels in the cell culture media obtained from the LTBMC assay. LTBMCs were established from the bone marrow of WT, Smad3-/-Fancd2+/+, Smad3+/+Fancd2-/- and Smad3 Fancd2-129B6F2 mice. The cell culture media was obtained after weeks 9 and 10 following establishment of the cultures and TGFβ levels were measured using a TGFβ ELISA kit. Plot shows the average of weeks 9 and 10. Smad3 Fancd2-129B6F2 cultures produce more TGFβ ligand than the cultures derived from other genotypes. Data are represented as mean ± SEM. p values of 0.01 to 0.05 were considered significant (*), (B) Western blots of the lysates from adult bone marrow stromal cells of Smad3 Fancd2-129B6F2 mice showing total Smad3 levels and total ERK levels after irradiation. Quantifications relative to wild type in basal conditions and the loading control are shown below every lane. Pink indicates the irradiated cultures per cell line. (C) Western blots of the lysates from adult bone marrow stromal cells of Smad3 Fancd2-129B6F2 mice showing SNAIL levels after irradiation. Quantifications relative to wild type in basal conditions and the loading control are shown below every lane. Pink indicates the irradiated cultures per cell line. (D) Western blots of the lysates from stromal cell lines showing the response of the canonical SMAD3 pathway and the non-canonical ERK pathway in cell lines derived from Smad3 Fancd2-129129F2 mice after irradiation. (E) Western blots of the lysates from stromal cell lines showing the response of the canonical SMAD3 pathway and the non-canonical ERK pathway in cell lines derived from Smad3 Fancd2-B6B6F2 mice after irradiation. (F) Western blots of the lysates from stroma cell lines showing the response of the canonical SMAD3 pathway and the non-canonical ERK pathway in cell lines derived from Smad3 Fancd2-B6129F2 mice after irradiation. (G) Representative plots of copy number variants (CNVs) across the genomes of representative embryos, containing denoised [file pgen.1010459.s004.tiff]

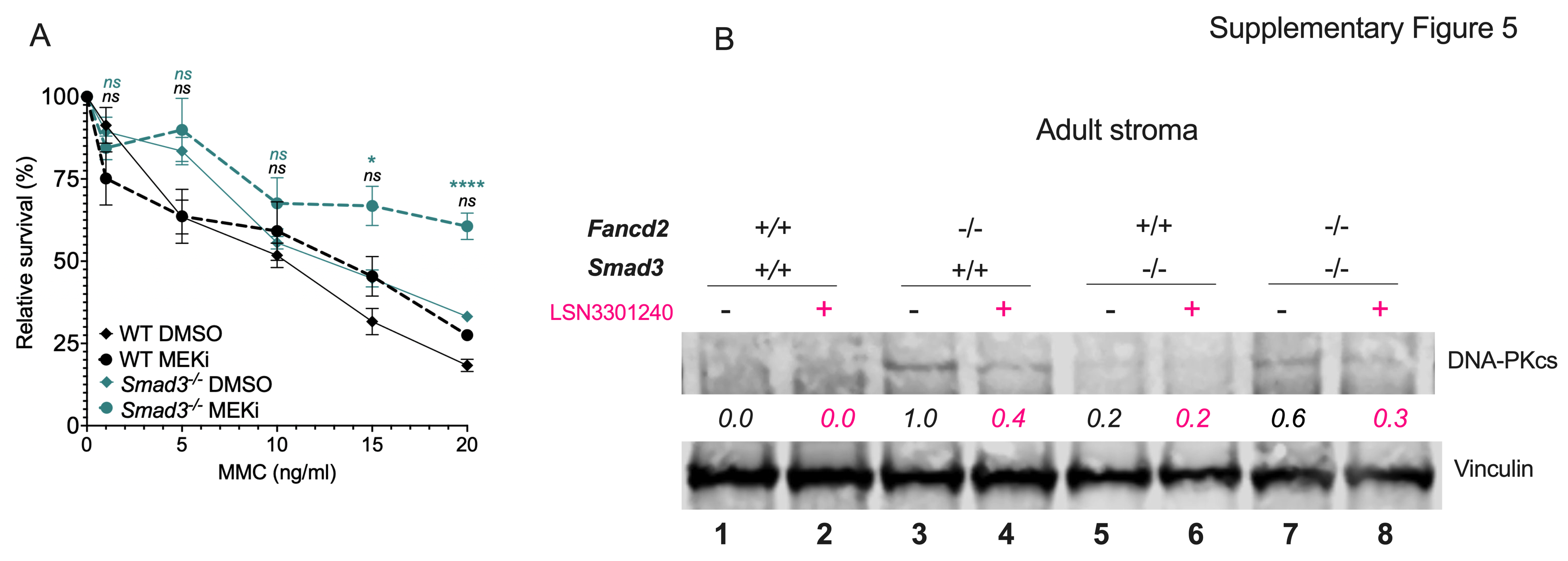

Supplement: S5 Fig — A) Survival curves of WT and Smad3-/- stromal cell lines in presence of MMC and the MEK inhibitor PD0325901. B) Western blots of the lysates from stromal cell lines showing that inhibition of TGFβ RI with LSN3301240 reduces the levels of DNA-PKcs in cells from both Fancd2-/- and Smad3 Fancd2-129B6F2 mice. A representative blot from two independent experiments is shown. Quantification relative to wild type in basal conditions and the loading control is shown below every lane. Pink indicates samples treated with LSN3301240. Data in (A) are represented as mean ± SEM. p values of 0.01 to 0.05 were considered significant (*), p values of 0.001 to 0.01 were considered very significant (**) and p values of < 0.001 were considered extremely significant (***, ****). (TIFF) [file pgen.1010459.s005.tiff]

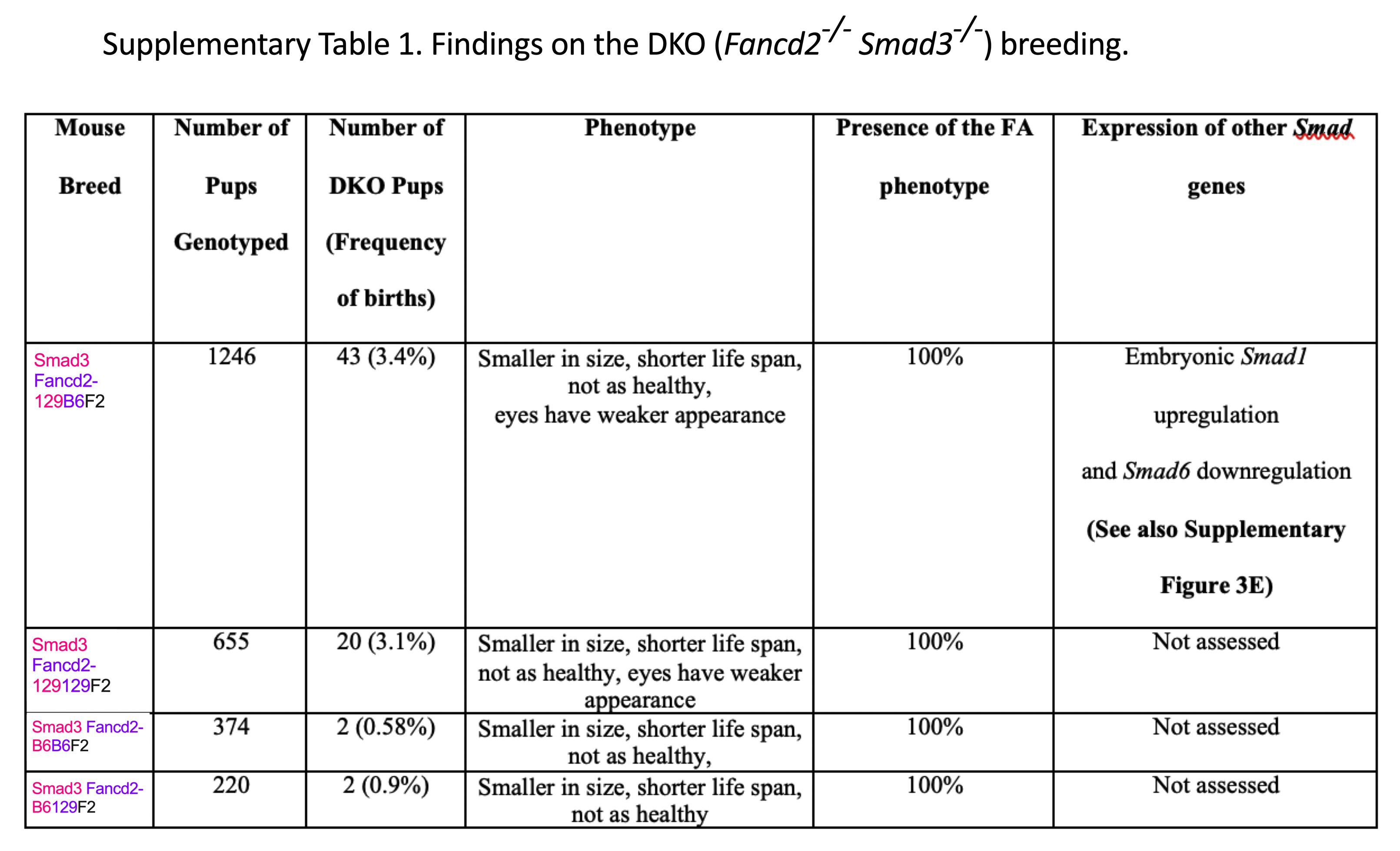

Supplement: S1 Table — This Table summarizes the phenotype observed in our DKO crosses. It can be observed variability in the number of pups and etention of the FA phenotype. (TIFF) [file pgen.1010459.s007.tiff]
